# Regulating metal–oxygen covalency in reconstructed sulfurized high-entropy perovskite to activate and stabilize lattice oxygen for the oxygen evolution reaction

**DOI:** 10.1039/d5sc04541j

**Published:** 2025-09-17

**Authors:** Xiang Li, Qiuju Li, Bingyu Chen, Mengna Wang, Chuanchuan Yan, Subhajit Jana, Ziqi Liao, Zhenyu Li, Dunfeng Gao, Guoxiong Wang

**Affiliations:** a State Key Laboratory of Catalysis Energy, Dalian National Laboratory for Clean Energy iChEM (Collaborative Innovation Center of Chemistry for Energy Materials), Dalian Institute of Chemical Physics Chinese Academy of Sciences Dalian 116023 China lizhenyu@dicp.ac.cn wanggx@dicp.ac.cn; b Dalian Jiaotong University Dalian 116028 China; c Department of Chemistry, College of Basic Medicine, Third Military Medical University (Army Medical University) Chongqing 400038 China; d University of Chinese Academy of Sciences Beijing 100049 China; e Department of Mechanical and Mechatronics Engineering, Waterloo Institute for Nanotechnology, Materials Interfaces Foundry, University of Waterloo Waterloo Ontario N2L3G1 Canada

## Abstract

Switching the adsorbate evolution mechanism (AEM) to the lattice oxygen mechanism (LOM) can break the theoretical limit of catalytic activity for the oxygen evolution reaction (OER). However, it is difficult for LOM-dominated catalysts to simultaneously obtain high activity and stability because of their trade-off relationship. Here, we report a reconstructed sulfurized high-entropy perovskite (S-LaNiFeCoCrMnO_3_), which possesses excellent activity with an overpotential of 165 mV and has a high catalytic stability for 1800 h at 10 mA cm^−2^ toward the OER. Furthermore, S-LaNiFeCoMnCrO_3_ as the anode catalyst in an anion exchange membrane water electrolyzer exhibits a high current density of 5.8 A cm^−2^ at a cell voltage of 2.0 V. On-line differential electrochemical mass spectrometry results suggest that the increased reactivity of lattice oxygen in reconstructed S-LaNiFeCoCrMnO_3_ facilitates the enhancement of OER activity. X-ray absorption near-edge structure and *in situ* Raman spectroscopy results reveal that the local Ni–S bond in the sulfurized layer on the surface of S-LaNiFeCoCrMnO_3_ drives the generation of the Fe–NiOOH active phase with a NiO_2_ subunit layer and high-valent Ni^4+^ species. Furthermore, strong covalent Ni–O and weak covalent Fe–O bonds in the Fe–NiOOH active phase play a critical role in activating and stabilizing lattice oxygen, thus breaking the activity–stability trade-off relationship for the LOM.

## Abstract 2

Promoting surface reconstruction for sulfurized high-entropy perovskite to form the Fe–NiOOH active phase with strong-covalent Ni–O bonds and weak-covalent Fe–O bonds, which exhibits industrial current density in an AEMWE.

## Graphical abstract

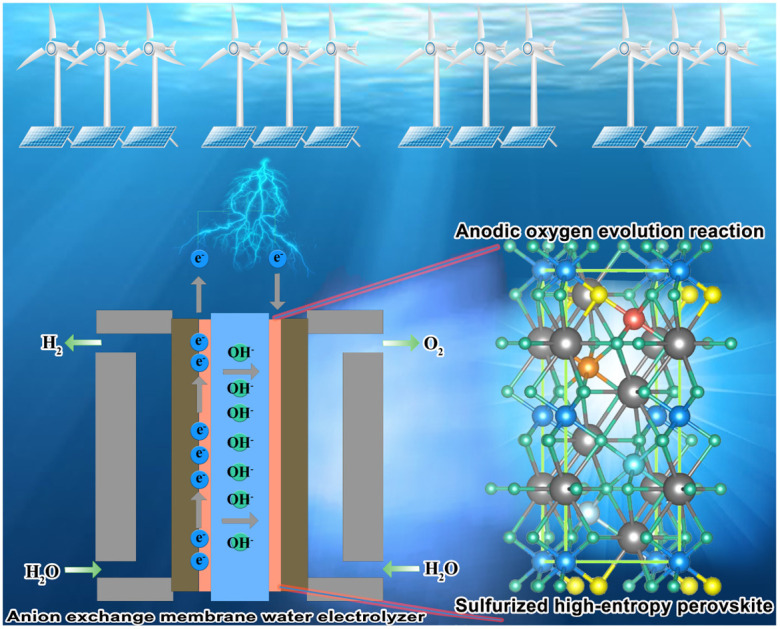

## Introduction

The growing global energy demand has accelerated the depletion of fossil fuels and generated serious environmental problems.^1,2^ Hydrogen as a typically clean and sustainable energy source is an important exploration direction for the future energy revolution that mankind should consider and conduct.^3,4^ Hydrogen production by using an anion exchange membrane water electrolyzer (AEMWE) has attracted much attention because it allows the application of nonprecious metal-based catalysts without affecting the catalytic activity and stability of the oxygen evolution reaction (OER).^5,6^ However, the slow kinetics of anodic OER leads to a high overpotential and excessive energy consumption, severely limiting the industrial process for the AEMWE.^7,8^ Therefore, exploring a high-performance OER catalyst is crucial for overcoming the high energy consumption in the AEMWE, and its first task is the in-depth understanding of the catalytic mechanism for the OER. In general, for the conventional adsorbate evolution mechanism (AEM), the adsorption strength of various intermediate adsorbents involved in the OER is highly proportional to their catalytic activity, and they also undergo a coordinated electron transfer process during electrocatalysis, resulting in a theoretical limit for the overpotential of 0.37 V.^9,10^ Unlike the AEM, the lattice oxygen mechanism (LOM) is a catalytic pathway of an uncoordinated proton-electron transfer process for directly coupling O–O bonds.^11,12^ Although the LOM-dominated catalysts break the theoretical overpotential limit of the AEM for the OER, it is still difficult to obtain satisfactory catalytic stability in a three-electrode system. Furthermore, it is more difficult to adapt the industrial water electrolysis operated in the AEMWE.

So far, several oxygen-containing OER catalysts, such as borates, spinels, hydroxides, and perovskites have been identified to conform to the LOM toward the OER.^13–15^ Among the above catalysts, due to the structural compatibility of various elements in the B position of perovskite oxides, high-entropy perovskite has emerged as an attractive candidate for the OER.^16^ Although the increased reactivity of lattice oxygen in high-entropy perovskite triggers the enhancement of catalytic activity toward the OER, the structural collapse by leaching metal cations during the electrocatalytic process results in poor stability. Furthermore, it is still difficult to be applied in AEMWEs because of the low conductivity.^17^ Therefore, it is crucial to regulate the lattice oxygen reactivity as well as solve the trade-off relationship between catalytic activity and stability for the LOM in high entropy perovskite. In addition, theoretical calculations and *in situ* spectroscopy studies have revealed that the oxide/hydroxide/oxyhydroxide formed on the catalyst surface by surface reconstruction during the OER facilitates the activation of lattice oxygen and plays a key role in the LOM pathway.^18,19^ While the previous studies have proved that self-restructuring during the OER is an effective way to obtain highly active LOM-based catalysts, the influences of composition and structure of the pre-catalyst on the catalytic activity have not been well investigated.^20^ This not only resulted in an incomplete understanding of the LOM, but also hindered the exploration and rational design of efficient OER catalysts. Regulating the covalency of the metal–oxygen bond in the LOM-based catalyst is favorable to the redox of lattice oxygen during the OER. Doping S with high electronegativity (2.58) not only increases the ion conductivity/oxygen ion diffusion rate on the perovskite surface, but also enhances the covalency of the metal–O bond, thus promoting the reactivity of lattice oxygen.^21^ Furthermore, introducing a sulfurized layer on the surface can facilitate surface reconstruction and significantly promote the generation of the active phase for oxide/hydroxide/oxyhydroxide on the high-entropy perovskite surface during the OER.^22^

Herein, a sulfurized high-entropy perovskite (S-LaNiFeCoCrMnO_3_) catalyst was prepared by a co-precipitation method and subsequent chemical vapor deposition process. The reconstructed S-LaNiFeCoCrMnO_3_ as an OER catalyst exhibited an overpotential of 165 mV and excellent catalytic stability for 1800 h. Furthermore, the reconstructed S-LaNiFeCoCrMnO_3_ as an anode catalyst in the AEMWE exhibited a current density of 1.0 A cm^−2^ and 5.8 A cm^−2^ at a cell voltage of 1.61 V and 2.0 V, respectively. Combining X-ray absorption near-edge structure spectroscopy (XANES), *in situ* Raman spectroscopy, and cyclic voltammetry (CV) results demonstrated that S atoms, which mainly coordinated with a Ni atom to form a Ni–S bond in the S-LaNiFeCoCrMnO_3_ pre-catalyst, were partially leached, increasing coordination numbers (CNs) of the Ni–O bond, promoting the adsorption of the oxygen-containing intermediates, and thus facilitating the surface reconstruction of the catalyst during the OER to *in situ* form the Fe–NiOOH active phase with a NiO_2_ subunit layer and Ni^4+^ species. On-line differential electrochemical mass spectrometry, TMAOH-distribution experiments, and density functional theory (DFT) calculation results reveal that the Fe–NiOOH active phase with a NiO_2_ subunit layer and Ni^4+^ species formed on the reconstructed S-LaNiFeCoCrMnO_3_ catalyst enhances the reactivity of lattice oxygen, thus improving the OER activity. Furthermore, strong covalent Ni–O and weak covalent Fe–O bonds in the Fe–NiOOH active phase played a critical role in activating and stabilizing lattice oxygen, thus breaking the trade-off relationship between activity and stability for the LOM.

## Results and discussion

### Synthesis and characterization of S-LaNiFeCoCrMnO_3_

**Fig. 1:**
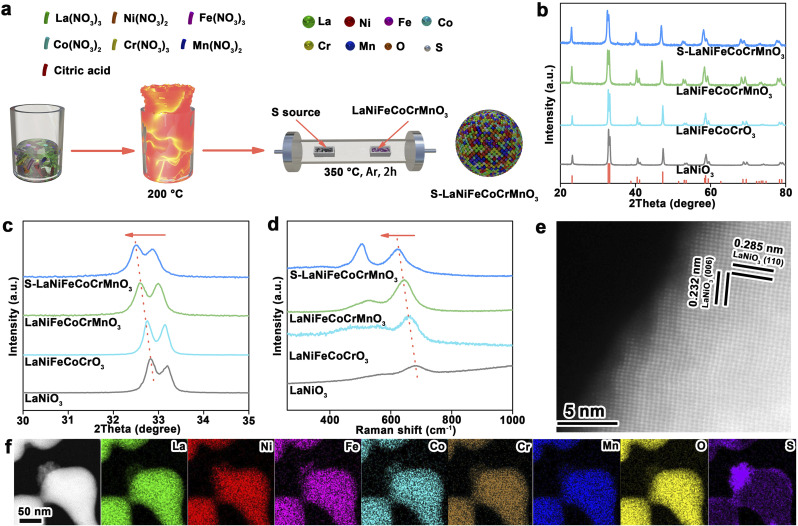
Synthesis and characterization of the S-LaNiFeCoCrMnO_3_ catalyst. (a) Synthesis schematic of the S-LaNiFeCoCrMnO_3_ catalyst. (b and c) XRD and corresponding magnified patterns of LaNiO_3_, LaNiFeCoCrO_3_, LaNiFeCoCrMnO_3_, and S-LaNiFeCoCrMnO_3_ catalysts. (d) Raman spectra of LaNiO_3_, LaNiFeCoCrO_3_, LaNiFeCoCrMnO_3_, and S-LaNiFeCoCrMnO_3_ catalysts. (e) HRTEM image of the S-LaNiFeCoCrMnO_3_ catalyst. (f) STEM image and corresponding elemental maps of the S-LaNiFeCoCrMnO_3_ catalyst.

A sulfurized high-entropy perovskite catalyst (S-LaNiFeCoCrMnO_3_) was synthesized through a two-step method (the detailed procedure was provided in the experimental section): (1) using the co-precipitation method to prepare a high-entropy perovskite oxide catalyst (LaNiFeCoCrMnO_3_); (2) subsequently, S-LaNiFeCoCrMnO_3_ was obtained using the chemical vapor deposition method to conduct the surface sulfuration of as-prepared LaNiFeCoCrMnO_3_ (Fig. 1a). X-ray diffraction (XRD) patterns demonstrate the precise synthesis of S-LaNiFeCoCrMnO_3_ with a single-phase hexagonal structure (Fig. 1b). The diffraction peak located at ∼33° for S-LaNiFeCoCrMnO_3_ shifts toward a low angle compared to that of LaNiO_3_, LaNiFeCoCrO_3_, and LaNiFeCoCrMnO_3_ in the magnified XRD patterns (Fig. 1c), indicative of the lattice distortions caused by the differences in atomic diameters of the six elements.^23^ The Raman spectra at ∼690 cm^−1^, representing *B*_1g_ stretching vibration of the octahedron (NiO_6_), showed a negatively shifted trend with the increase in the number of elements in the crystal structure for LaNiO_3_, LaNiFeCoCrO_3_, LaNiFeCoCrMnO_3_, and S-LaNiFeCoCrMnO_3_ catalysts (Fig. 1d), indicating the enhanced degree of lattice distortion.^24^ Besides, the strong Raman peak, which appeared at ∼500 cm^−1^, is ascribed to the NiO_6_ octahedron for S-LaNiFeCoCrMnO_3_. The introduction of S in S-LaNiFeCoCrMnO_3_ leads to an increased Ni^3+^ ratio, thus enhancing Raman peak intensity of NiO_6_. Transmission electron microscopy (TEM) and scanning electron microscopy (SEM) images show that S-LaNiFeCoCrMnO_3_ has a similar morphology of nanoparticles with a size of 100–200 nm compared to LaNiO_3_, LaNiFeCoCrO_3_, and LaNiFeCoCrMnO_3_ (Fig. S1 and S2). Different from LaNiO_3_, LaNiFeCoCrO_3_, and LaNiFeCoCrMnO_3_, a rough surface can be observed for S-LaNiFeCoCrMnO_3_. High-resolution TEM (HRTEM) displays the measured interplanar distances of 0.232 nm and 0.285 nm, assigned to the (006) and (110) crystal facets for S-LaNiFeCoCrMnO_3_, respectively (Fig. 1e). Besides, the (110) interplanar distance in S-LaNiFeCoCrMnO_3_ is larger than that in LaNiO_3_, LaNiFeCoCrO_3_, and LaNiFeCoCrMnO_3_ (Fig. 1e and S3), demonstrating the lattice expansion. STEM and SEM elemental maps show that La, Ni, Fe, Co, Cr, Mn, and O elements are uniformly distributed in S-LaNiFeCoCrMnO_3_ while the S element is mainly distributed on its surface overlayer, demonstrating that a thin sulfurized layer forms on the surface of S-LaNiFeCoCrMnO_3_ (Fig. 1f and S4).

### OER performance of S-LaNiFeCoCrMnO_3_

**Fig. 2:**
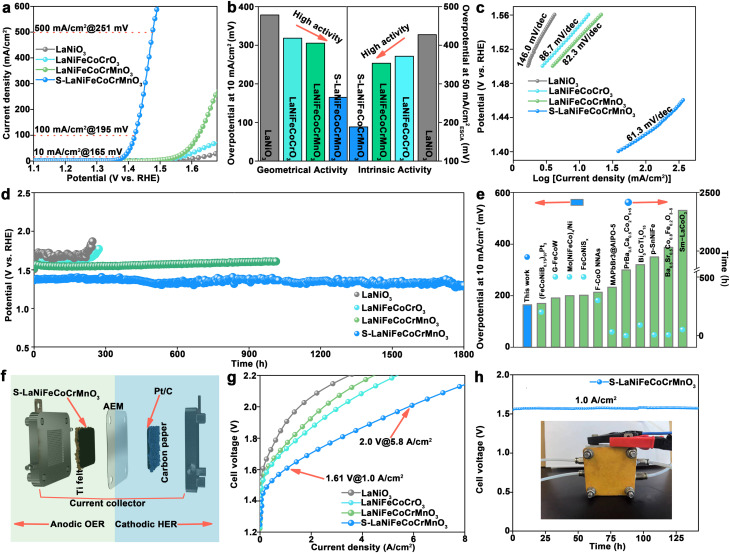
Electrocatalytic performance evaluation of the S-LaNiFeCoCrMnO_3_ catalyst toward the OER in 1.0 M KOH solution. (a) LSV curves of LaNiO_3_, LaNiFeCoCrO_3_, LaNiFeCoCrMnO_3_, and S-LaNiFeCoCrMnO_3_ catalysts. (b) Comparison of geometric and ECSA-normalized activity for LaNiO_3_, LaNiFeCoCrO_3_, LaNiFeCoCrMnO_3_, and S-LaNiFeCoCrMnO_3_ catalysts. (c) Tafel slopes of LaNiO_3_, LaNiFeCoCrO_3_, LaNiFeCoCrMnO_3_, and S-LaNiFeCoCrMnO_3_ catalysts. (d) Chronopotentiometric curves of LaNiO_3_, LaNiFeCoCrO_3_, LaNiFeCoCrMnO_3_, and S-LaNiFeCoCrMnO_3_ catalysts at 10 mA cm^−2^. (e) Comparison of S-LaNiFeCoCrMnO_3_ and previously reported catalysts for perovskite oxides and sulfides. (f) Schematic diagram to illustrate the anodic oxygen evolution and cathodic hydrogen evolution in AEMWEs. (g) Polarization curves of LaNiO_3_, LaNiFeCoCrO_3_, LaNiFeCoCrMnO_3_, and S-LaNiFeCoCrMnO_3_ as anode catalysts in an anion exchange membrane water electrolyzer. (h) Chronopotentiometric curve of S-LaNiFeCoCrMnO_3_ as an anode catalyst at 1.0 A cm^−2^ in an AEMWE.

We conducted the electrochemical measurements to evaluate the catalytic performance toward the OER in 1.0 M KOH using a standard three-electrode system. Linear sweep voltammetry (LSV) normalized by using electrode area in Fig. 2a shows that S-LaNiFeCoCrMnO_3_ can obtain a current density of 10 mA cm^−2^ at an overpotential of 165 mV, outperforming LaNiO_3_ (389 mV), LaNiFeCoCrO_3_ (329 mV), and LaNiFeCoCrMnO_3_ (315 mV). Impressively, S-LaNiFeCoCrMnO_3_ only needs an overpotential of 195 mV and 251 mV to gain a high current density of 100 and 500 mA cm^−2^, respectively. The electrochemical active surface area (ECSA) of S-LaNiFeCoCrMnO_3_ is significantly higher than that of LaNiO_3_, LaNiFeCoCrO_3_, and LaNiFeCoCrMnO_3_, demonstrating the exposure of more active sites after sulfuration (Fig. S5 and S6). Furthermore, the ECSA-normalized catalytic performance exhibits an increased trend in the order of LaNiO_3_, LaNiFeCoCrO_3_, LaNiFeCoCrMnO_3_, and S-LaNiFeCoCrMnO_3_, confirming the same trend as their geometric activities (Fig. 2b and S7). S-LaNiFeCoCrMnO_3_ possesses superior activity when the reaction time and temperature in the synthesis procedure were set to 2 h and 350 °C, respectively (Fig. S8–S11). As shown in Fig. 2c, S-LaNiFeCoCrMnO_3_ has the smallest Tafel slope of 61.3 mV dec^−1^ compared to LaNiO_3_ (146.0 mV dec^−1^), LaNiFeCoCrO_3_ (86.7 mV dec^−1^), and LaNiFeCoCrMnO_3_ (82.3 mV dec^−1^), accelerating the reaction kinetics toward the OER.^25,26^ The electrochemical impedance spectroscopy (EIS) test reveals that electron-transfer resistance of S-LaNiFeCoCrMnO_3_ is lower than that of LaNiO_3_, LaNiFeCoCrO_3_, and LaNiFeCoCrMnO_3_, demonstrating fast charge-transfer capacity at the interface between the catalyst and electrolyte for facilitating the reaction kinetics toward the OER (Fig. S12).^27^ In addition, S-LaNiFeCoCrMnO_3_ exhibits a faradaic efficiency approaching 100% (Fig. S13).

The stability of the catalyst is an important index for the OER. As shown in Fig. 2d, the overpotentials at 10 mA cm^−2^ toward the OER for LaNiO_3_ and LaNiFeCoCrO_3_ display a pronounced increase before 300 h. Compared with LaNiO_3_ and LaNiFeCoCrO_3_, the overpotential of LaNiFeCoCrMnO_3_ exhibits less of an increase before 300 h, but shows a slight increase after ∼1000 h, demonstrating that the regulation of entropy in pre-catalysts can enhance the stability toward the OER. In particular, S-LaNiFeCoCrMnO_3_ maintains excellent stability for 1800 h (75 days), confirming that the sulfurized overlayer formed on S-LaNiFeCoCrMnO_3_ further enhances the stability of the catalyst. As for S-LaNiFeCoCrMnO_3_, the activity is increased at the initial stage before 600 h during the stability test, suggesting the occurrence of surface reconstruction. Besides, S-LaNiFeCoCrMnO_3_ exhibits a more prolonged surface reconstruction than that of LaNiO_3_, LaNiFeCoCrO_3_, and LaNiFeCoCrMnO_3_, suggesting that the existence of a sulfide layer promotes surface reconstruction. After surface reconstruction, the activity of S-LaNiFeCoCrMnO_3_ remains stable, implying that the lattice oxygen participates in the OER. The XRD pattern and SEM image after the stability test show that the morphology, phase structure, and elemental distribution are unchanged, demonstrating excellent structural stability of S-LaNiFeCoCrMnO_3_ (Fig. S14–S16). Furthermore, inductively coupled plasma-optical emission spectrometry (ICP-OES) result reveals that the La, Ni, Fe, Co, Cr, and Mn elements did not dissolve out obviously, while S precipitated obviously for the S-LaNiFeCoCrMnO_3_ catalyst during the OER (Fig. S17). S leaching in S-LaNiFeCoCrMnO_3_ during the OER increases coordination numbers (CNs) of the Ni–O bond, promoting the adsorption of the oxygen-containing intermediates, and thus facilitating the surface reconstruction of the catalyst during the OER to *in situ* form the oxyhydroxide active phase. Considering the low overpotential and high stability, S-LaNiFeCoCrMnO_3_ possesses higher OER performance than most previously reported catalysts, including perovskites, sulfides, and even other high-entropy materials (Table S1 and Fig. 2e).^28–30^

To evaluate the industrial prospects of the S-LaNiFeCoCrMnO_3_ catalyst, we conducted the AEMWE measurements. S-LaNiFeCoCrMnO_3_ as an anode catalyst was coated on the side of the Ti felt gas diffusion layer (GDL), while commercial Pt/C as a cathode catalyst was coated on the carbon paper GDL. Subsequently, the Ti felt GDL with S-LaNiFeCoCrMnO_3_ and carbon paper GDL with Pt/C were pressed on both sides of an AEM, respectively, thus establishing membrane electrode assembly (MEA) for the AEMWE (Fig. 2f). The SEM image (Fig. S18) and corresponding cross-sectional SEM elemental maps (Fig. S19) illustrate that the thickness of the catalyst layer on the S-LaNiFeCoCrMnO_3_-coated GDL is ∼10 μm. Polarization curves of AEMWEs measured in 1.0 M KOH at 80 °C show that S-LaNiFeCoCrMnO_3_ as an anode catalyst only requires a cell voltage of 1.61 V to deliver a current density of 1.0 A cm^−2^, outperforming LaNiFeCoCrMnO_3_ (1.73 V), LaNiFeCoCrO_3_ (1.76 V), LaNiO_3_ (1.89 V), and previously reported representative catalysts (Fig. 2g and Table S2). Moreover, the high current density of 5.8 A cm^−2^ can be obtained at a cell voltage of 2.0 V, suggesting the industrial prospect of S-LaNiFeCoCrMnO_3_. The AEMWE using the S-LaNiFeCoCrMnO_3_ anode catalyst can steadily operate at a current density of 1.0 A cm^−2^ for over 140 h, suggesting excellent catalytic stability under the industrial conditions (Fig. 2h). The SEM image and corresponding elemental maps reveal that the catalyst layer still integrally existed on the AEM surface after the stability test, suggesting the anti-corrosion capacity (Fig. S20).

### Revealing the active phase for the S-LaNiFeCoCrMnO_3_ catalyst

**Fig. 3:**
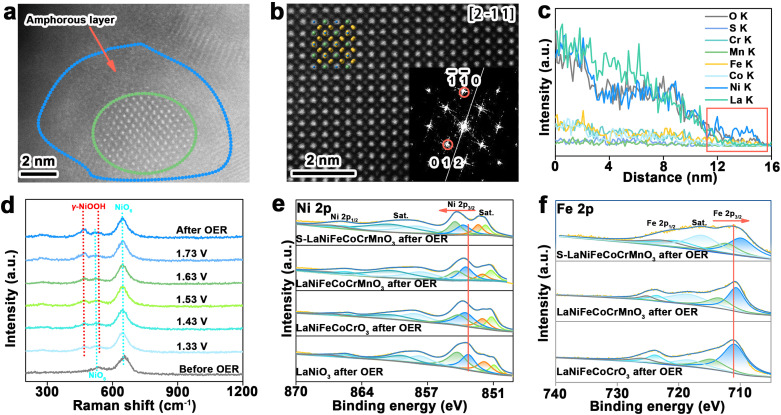
Recognition of the active phase for the S-LaNiFeCoCrMnO_3_ catalyst toward the OER. (a) High-resolution HAADF-STEM image of the S-LaNiFeCoCrMnO_3_ catalyst after the stability test. (b) Magnified high-resolution HAADF-STEM image of the S-LaNiFeCoCrMnO_3_ catalyst after the stability test. The inset shows the corresponding FFT image. (c) Atom-level line scanning EDX spectra of the S-LaNiFeCoCrMnO_3_ catalyst after the stability test. (d) *In situ* Raman spectra of the S-LaNiFeCoCrMnO_3_ catalyst. (e) Ni 2p XPS spectra of LaNiO_3_, LaNiFeCoCrO_3_, LaNiFeCoCrMnO_3_, and S-LaNiFeCoCrMnO_3_ catalysts after the stability test. (f) Fe 2p XPS spectra of LaNiFeCoCrO_3_, LaNiFeCoCrMnO_3_, and S-LaNiFeCoCrMnO_3_ catalysts after the stability test.

To reveal the catalytic active phase of S-LaNiFeCoCrMnO_3_, X-ray photoelectron spectroscopy (XPS), aberration-corrected high angle annular dark field-scanning transmission electron microscopy (HAADF-STEM), and *in situ* Raman spectroscopy measurements were carried out. The HAADF-STEM image shows that the surface yields an amorphous oxide layer with a thickness of 2 nm (Fig. 3a), suggesting the reconstruction of the S-LaNiFeCoCrMnO_3_ surface after the OER. The HAADF-STEM image also shows that the atomic arrangement and the resultant fast Fourier transform (FFT) pattern of the S-LaNiFeCoCrMnO_3_ catalyst after the stability test are consistent with the corresponding theoretical result, indicating that it still maintains the original structure of the bulk phase (Fig. 3b, S21 and S22). Atomic-level line scanning EDX spectra from bulk to the surface show that the amorphous oxide layer on the S-LaNiFeCoCrMnO_3_ surface mainly contains Ni, Fe, and O elements (Fig. S23 and 3c).

*In situ* Raman spectra for LaNiO_3_, LaNiFeCoCrO_3_, LaNiFeCoCrMnO_3_, and S-LaNiFeCoCrMnO_3_ catalysts are shown in Fig. 3d and S24. For LaNiO_3_, LaNiFeCoCrO_3_, and LaNiFeCoCrMnO_3_ catalysts, a typical characteristic peak of LaNiO_3_ at 402 cm^−1^ is observed during the OER. Unlike the LaNiO_3_, LaNiFeCoCrO_3_, and LaNiFeCoCrMnO_3_ catalysts, the characteristic peak of LaNiO_3_ for the S-LaNiFeCoCrMnO_3_ catalyst is absent, suggesting that the existence of the sulfurized layer on the S-LaNiFeCoMnCrO_3_ surface buries the signal of LaNiO_3_. Besides, two peaks at 472 and 542 cm^−1^, assigned to γ-NiOOH with a NiO_2_ subunit layer, respectively, appeared on LaNiFeCoCrO_3_ and LaNiFeCoCrMnO_3_ catalysts until the potential of 1.53 V *vs.* RHE was applied.^31^ However, the peaks of γ-NiOOH didn't appear on LaNiO_3_. Furthermore, the emergence of the peaks for γ-NiOOH on S-LaNiFeCoMnCrO_3_ (1.33 V) is much earlier than on LaNiFeCoCrO_3_ and LaNiFeCoCrMnO_3_ catalysts. This result suggests that the existence of the sulfurized layer accelerates the surface reconstruction and facilitates the formation of the γ-NiOOH active phase with a NiO_2_ subunit layer, thus enhancing the reactivity of lattice oxygen toward the OER. Coupling the *in situ* Raman spectra and HAADF-STEM results, it is proposed that the *in situ* formed Fe–NiOOH with a NiO_2_ subunit layer during the OER is the catalytic active phase toward the OER.^32^

XPS spectra of Ni 2p for LaNiO_3_, LaNiFeCoCrO_3_, LaNiFeCoCrMnO_3_, and S-LaNiFeCoCrMnO_3_ catalysts after the stability test in Fig. 3e show the binding energies of Ni^3+^ 2p_3/2_ and Ni^3+^ 2p_1/2_ located at 854.4 eV and 865.7 eV, respectively. In Fig. 3f, there are two obvious peaks at 715.0 eV and 726.2 eV, which are attributed to the binding energy of Fe^3+^ 2p_3/2_ and Fe^3+^ 2p_1/2_, respectively. With the increase in elements/entropy in the four perovskites, the binding energies of Ni^3+^ 2p_3/2_ exhibit a positive shift trend in the order of LaNiO_3_, LaNiFeCoCrO_3_, LaNiFeCoCrMnO_3_, and S-LaNiFeCoCrMnO_3_ after the stability test, whereas the binding energies of Fe^3+^ 2p_3/2_ exhibit a negative shift trend in the order of LaNiFeCoCrO_3_, LaNiFeCoCrMnO_3_, and S-LaNiFeCoCrMnO_3_ after the stability test. Furthermore, the binding energies of Co^3+^ 2p_3/2_, Cr^3+^ 2p_3/2_, and Mn^3+^ 2p_3/2_ also display a decreased trend with the increase in elements in LaNiFeCoCrO_3_, LaNiFeCoCrMnO_3_, and S-LaNiFeCoCrMnO_3_ catalysts (Fig. S25). This result indicates the strong electron transfer between Ni as the electron donor and Fe, Co, Cr, and Mn as the electron acceptors in S-LaNiFeCoCrMnO_3_, which facilitates the generation of high-valent Ni for *in situ* formed Fe–NiOOH during the OER.^33^ CV curves of the catalysts in Fig. S26 show that S-LaNiFeCoCrMnO_3_ possesses more positive potential of Ni^3+^/Ni^4+^ redox peaks (1432 V) compared with that of LaNiO_3_ (1.341 V), LaNiFeCoCrO_3_ (1.353 V), and LaNiFeCoCrMnO_3_ (1.395 V), demonstrating the formation of high-valent Ni^4+^ species in the NiOOH active phase, formed on reconstructed S-LaNiFeCoCrMnO_3_ during the OER because the existence of the sulfurized layer on the S-LaNiFeCoMnCrO_3_ surface promotes the surface reconstruction.^34,35^ Therefore, we demonstrate that the *in situ* formed Fe–NiOOH active phase with a NiO_2_ subunit layer on the reconstructed S-LaNiFeCoMnCrO_3_ catalyst possesses high-valent Ni^4+^ species. The high-valent Ni^4+^ in S-LaNiFeCoCrMnO_3_ also further results in the enhancement of the covalency of the Ni-oxygen bond, thus reinforcing the reactivity of lattice oxygen in the LOM.^36^

### Mechanism insight on S-LaNiFeCoCrMnO_3_ for the OER

**Fig. 4:**
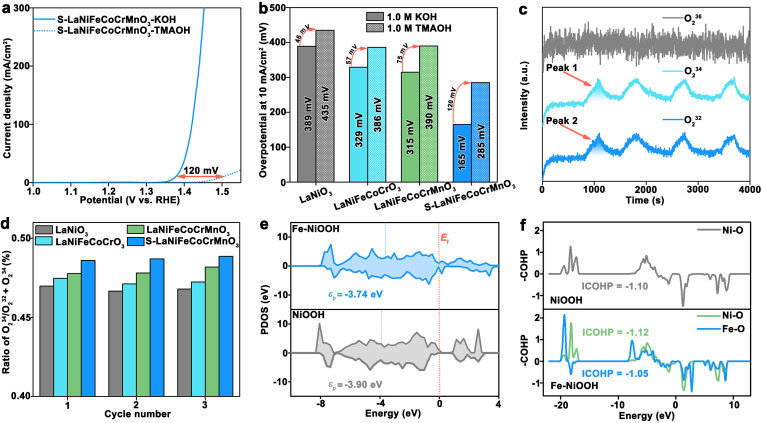
Expounding the LOM for the S-LaNiFeCoCrMnO_3_ catalyst toward the OER. (a) LSV curves of the S-LaNiFeCoCrMnO_3_ catalyst in 1.0 M KOH and 1.0 M TMAOH. (b) Overpotential comparison of LaNiO_3_, LaNiFeCoCrO_3_, LaNiFeCoCrMnO_3_, and S-LaNiFeCoCrMnO_3_ catalysts in 1.0 M KOH and 1.0 M TMAOH. (c) DEMS signals of O_2_ products for the ^18^O-labeled S-LaNiFeCoCrMnO_3_ catalyst in 1.0 M KOH with H_2_^16^O. (d) Comparison of the peak area ratio of ^34^O_2_/(^34^O_2_ + ^32^O_2_) for LaNiO_3_, LaNiFeCoCrO_3_, LaNiFeCoCrMnO_3_, and S-LaNiFeCoCrMnO_3_ catalysts. (e) PDOS of lattice O atoms in NiOOH and Fe–NiOOH. (f) COHP plots of the Ni–O bond for NiOOH and Fe–O and Ni–O bonds for Fe–NiOOH.

In general, the OER mechanism mainly includes the adsorbate evolution mechanism (AEM) pathway and lattice oxygen mechanism (LOM) pathway. The AEM undergoes four concerted proton-electron transfer steps (CPET) occurring on metal sites, while the LOM proceeds through a non-concerted proton-electron transfer process. Unlike the AEM pathway, the O_2_^2−^ forms for the LOM pathway during the OER. Therefore, the detection of O_2_^2−^ species during the OER can effectively identify the reactivity of lattice oxygen for the LOM. The catalytic activity of the catalyst and corresponding kinetics toward the OER would reduce when a tetramethylammonium cation (TMA^+^) attacks the O_2_^2−^ species. As shown in Fig. 4a, the S-LaNiFeCoCrMnO_3_ catalyst exhibits significantly decreased OER overpotential in TMAOH solution than in KOH solution at 10 mA cm^−2^. The difference of overpotential in 1.0 M TMAOH and 1.0 M KOH for S-LaNiFeCoCrMnO_3_ (120 mV) is higher than that of LaNiO_3_ (46 mV), LaNiFeCoCrO_3_ (57 mV), and LaNiFeCoCrMnO_3_ (75 mV), suggesting the enhanced reactivity of lattice oxygen for S-LaNiFeCoCrMnO_3_ (Fig. 4b and S27).^37^

To further evaluate the reactivity of lattice oxygen for S-LaNiFeCoCrMnO_3_, on-line differential electrochemical mass spectrometry (DEMS) experiments were conducted in 1.0 M KOH solution with H_2_^16^O by using ^18^O isotope-labeled catalysts, including LaNiO_3_, LaNiFeCoCrO_3_, LaNiFeCoCrMnO_3_, and S-LaNiFeCoCrMnO_3_ (Fig. S28). The DEMS results in Fig. 4c and S29 reveal that the signals of O_2_^32^ and O_2_^34^ were detected, suggesting the generation of O^18^O^16^ species during the OER. Simultaneously, this result also further identifies that they follow the LOM. In addition, the ratio of O_2_^34^ to (O_2_^32^ + O_2_^34^) shows an increased trend in the order of LaNiO_3_, LaNiFeCoCrO_3_, LaNiFeCoCrMnO_3_, and S-LaNiFeCoCrMnO_3_, implying that the lattice oxygen in the S-LaNiFeCoCrMnO_3_ catalyst more actively participated in the OER process (Fig. 4d).^38^

DFT calculations were employed to investigate reaction mechanisms and activity origin. The Fe–NiOOH and NiOOH models were constructed to simulate the surface active phase on reconstructed S-LaNiFeCoCrMnO_3_ and LaNiO_3_ toward the OER (Fig. S30). Partial density of states (PDOS) results of lattice O atoms in Fig. 4e showed that 2p orbitals of O atoms for Fe–NiOOH have a higher p-band center (*ε*_p_) (−3.74 eV) than NiOOH (−3.90 eV), indicative of a higher lattice O activity, thereby enhancing the ratio of the LOM during the OER.^39–43^ Furthermore, integrated crystal orbital overlap population (ICOHP) of Fe–O bonds in Fe–NiOOH (−1.05) was more positive than that of Ni–O bonds in Fe–NiOOH (−1.12) and NiOOH (−1.10), indicating the weakened Fe–O bonds and strong Ni–O bonds (Fig. 4f).^44^ Hence, the introduction of an Fe component enhances the Ni–O covalent bond, promoting the activity of lattice oxygen in Fe–NiOOH. The electron localization function (ELF) of Fe–NiOOH and NiOOH was further calculated to investigate the covalency of Ni–O and Fe–O bonds. As shown in Fig. S31, Ni–O bonds in NiOOH have an ELF value of 0.72, while Ni–O bonds in Fe–NiOOH possess ELF values of 0.83, respectively. An ELF closer to 1 means a stronger covalency. Hence, the introduction of Fe in the Fe–NiOOH active phase enhances the Ni–O covalent bond, thereby promoting the lattice O activity.^45^

### Investigating atomic structure information for reconstructed S-LaNiFeCoCrMnO_3_

**Fig. 5:**
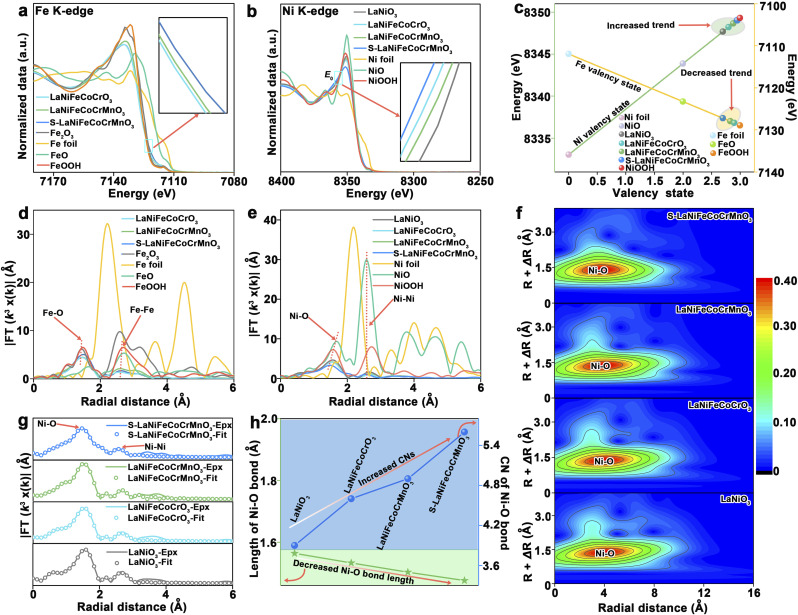
Atomic structural information for reconstructed S-LaNiFeCoCrMnO_3_ catalyst toward the OER. (a) Fe K-edge XANES of LaNiFeCoCrO_3_, LaNiFeCoCrMnO_3_, and S-LaNiFeCoCrMnO_3_ catalysts after the stability test, and Fe foil, FeO, Fe_2_O_3_, and FeOOH act as the reference samples. (b) Ni K-edge XANES of LaNiO_3_, LaNiFeCoCrO_3_, LaNiFeCoCrMnO_3_, and S-LaNiFeCoCrMnO_3_ catalysts after the stability test, and Ni foil, NiO, and NiOOH act as the reference samples. (c) Valence information of LaNiO_3_, LaNiFeCoCrO_3_, LaNiFeCoCrMnO_3_, and S-LaNiFeCoCrMnO_3_ catalysts after the stability test. (d) Fe K-edge FT-XANES of LaNiFeCoCrO_3_, LaNiFeCoCrMnO_3_, and S-LaNiFeCoCrMnO_3_ catalysts after the stability test, and Fe foil FeO, Fe_2_O_3_, and FeOOH act as the reference samples. (e) Ni K-edge FT-XANES of LaNiO_3_, LaNiFeCoCrO_3_, LaNiFeCoCrMnO_3_, and S-LaNiFeCoCrMnO_3_ catalysts after the stability test, and Ni foil, NiO, and NiOOH act as the reference samples. (f) Ni K-edge WT-XANES of LaNiO_3_, LaNiFeCoCrO_3_, LaNiFeCoCrMnO_3_, and S-LaNiFeCoCrMnO_3_ catalysts after the stability test. (g) Fitting data of Ni K-edge FT-XANES of LaNiO_3_, LaNiFeCoCrO_3_, LaNiFeCoCrMnO_3_, and S-LaNiFeCoCrMnO_3_ catalysts after the stability test. (h) Variational trend of Ni–O bond CNs and length for LaNiO_3_, LaNiFeCoCrO_3_, LaNiFeCoCrMnO_3_, and S-LaNiFeCoCrMnO_3_ catalysts after the stability test.

X-ray absorption near-edge structure (XANES) was further used to investigate the structural and valence state change of reconstructed LaNiO_3_, LaNiFeCoCrO_3_, LaNiFeCoCrMnO_3_, and S-LaNiFeCoCrMnO_3_ catalysts after the stability test. As shown in Fig. 5a, the Fe-edge XANES spectra show that the adsorption edge position exhibits a decreased trend in the order of reconstructed LaNiFeCoCrO_3_, LaNiFeCoCrMnO_3_, and S-LaNiFeCoCrMnO_3_. This demonstrates that the valence state of Fe in reconstructed S-LaNiFeCoCrMnO_3_ is lower than that of reconstructed LaNiFeCoCrO_3_ and LaNiFeCoCrMnO_3_, even if Fe–NiOOH *in situ* formed on the catalyst surface.^46^ The Ni K-edge XANES spectra of reconstructed LaNiO_3_, LaNiFeCoCrO_3_, LaNiFeCoCrMnO_3_, and S-LaNiFeCoCrMnO_3_ after the stability test are shown in Fig. 5b. The white-edge energy position of the Ni K-edge for S-LaNiFeCoCrMnO_3_ after the stability test is lower than that for LaNiO_3_, LaNiFeCoCrO_3_, and LaNiFeCoCrMnO_3_. This result demonstrates that the oxidation state for Ni species of reconstructed S-LaNiFeCoCrMnO_3_ after the stability test is much closer to that of NiOOH compared with that of reconstructed LaNiO_3_, LaNiFeCoCrO_3_, and LaNiFeCoCrMnO_3_, indicating the formation of a higher Ni oxidation state.

As shown in Fig. 5c, the average Ni oxidation state of reconstructed LaNiO_3_, LaNiFeCoCrO_3_, LaNiFeCoCrMnO_3_, and S-LaNiFeCoCrMnO_3_ exhibits an increased trend, while the average Fe oxidation state of reconstructed LaNiFeCoCrO_3_, LaNiFeCoCrMnO_3_, and S-LaNiFeCoCrMnO_3_ exhibits a decreased trend. This result demonstrates that the Ni and Fe acted as the electron donor and acceptor in the *in situ* formed Fe–NiOOH on the catalyst surface after the OER, respectively, promoting the electron transfer and the formation of Ni active sites with high valency. Fourier-transformed XANES (FT-XANES) spectra in Fig. 5d exhibit two dominant peaks at ∼1.44 Å and ∼2.57 Å, assigned to the Fe–O bond and Fe–Fe bond, respectively.^47^ The increased Fe–O bond length of S-LaNiFeCoCrMnO_3_ compared with LaNiFeCoCrO_3_ and LaNiFeCoCrMnO_3_ indicates the weakened metal–oxygen bond covalency, enhancing the stability of lattice oxygen during the OER (Fig. S32).^48^ The wavelet-transformed (WT) XANES (WT-XANES) analysis results further support the above FT-XANES result (Fig. S33 and S34). The fitted data of FT-XANES reveal that the CNs of the Fe–O bond for reconstructed S-LaNiFeCoCrMnO_3_ is significantly higher than that of reconstructed LaNiFeCoCrO_3_ and LaNiFeCoCrMnO_3_ catalysts, indicating that it possesses more space for adsorbing the oxygen-containing intermediates and further enhancing the catalytic performance (Fig. S33 and Table S3).^27^

FT-XANES spectra show that the Ni–O (∼1.58 Å) and Ni–Ni (∼2.73 Å) bonds were observed on LaNiO_3_, LaNiFeCoCrO_3_, LaNiFeCoCrMnO_3_, and S-LaNiFeCoCrMnO_3_ (Fig. 5e).^49^ The WT-XANES analysis result is consistent with the above FT-XANES results (Fig. 5f). The significant decrease in the Ni–O bond length of reconstructed S-LaNiFeCoCrMnO_3_ compared with that of reconstructed LaNiO_3_, LaNiFeCoCrO_3_, and LaNiFeCoCrMnO_3_ suggests the enhanced metal–O covalency, thus reinforcing the reactivity of lattice oxygen around the Ni site in the *in situ* formed Fe–NiOOH during the OER (Fig. 5g). Besides, XANES spectra of Ni, Co, and Fe K-edge reveal that the Ni–S bond rather than Fe–S and Co–S bonds was observed before the OER, suggesting that the S atoms coordinated with the Ni atoms on the S-LaNiFeCoCrMnO_3_ catalyst surface (Fig. S35–S37 and Tables S4–S6), whereas the intensity of the Ni–S bond for S-LaNiFeCoCrMnO_3_ reduced after the OER, which is consistent with ICP-OES results (Fig. S17). This result can infer that the existence of the Ni–S bond in S-LaNiFeCoCrMnO_3_ facilitates the surface reconstruction for *in situ* formation of the Fe–NiOOH active phase during the OER. Furthermore, as the number of doped elements for the original perovskite is increased, the Ni–O bond length and CNs exhibit the decreased and increased trend in the order of LaNiO_3_, LaNiFeCoCrO_3_, LaNiFeCoCrMnO_3_, and S-LaNiFeCoCrMnO_3_ after the stability test for the OER, respectively (Fig. 5h, S38, and Table S7). The high CNs of the Ni–O bond in S-LaNiFeCoCrMnO_3_ after the OER imply that the *in situ* formed Fe–NiOOH on the catalyst surface could provide more space to bind oxygen-containing intermediates for improving the OER activity. Furthermore, CNs and the length of the Ni–O bond for LaNiO_3_, LaNiFeCoCrO_3_, LaNiFeCoCrMnO_3_, and S-LaNiFeCoCrMnO_3_ after the stability test are close to those of the NiOOH reference rather than NiO, further demonstrating the *in situ* formation of the Fe–NiOOH active phase. Therefore, from the above XANES result, we can summarize the following points: (i) S atoms, which mainly coordinated with a Ni atom to form a Ni–S bond in the S-LaNiFeCoCrMnO_3_ pre-catalyst, are partially leached, increasing CNs of the Ni–O bond, promoting the adsorption of the oxygen-containing intermediates, and thus facilitating the surface reconstruction of the pre-catalyst during the OER to *in situ* form the Fe–NiOOH active phase with a NiO_2_ subunit layer and Ni^4+^ species. Ni as the electron donor and Fe, Co, Cr, and Mn as the electron acceptors in the S-LaNiFeCoCrMnO_3_, which facilitate the generation of high-valent Ni^4+^ for *in situ* formed Fe–NiOOH with a NiO_2_ layer during the OER.; (ii) the reduced Ni–O bond length implies that the reactivity of lattice oxygen located around the Ni sites was activated, while the enhanced Fe–O bond length means that the lattice oxygen near the Fe was stabilized for reconstructed S-LaNiFeCoCrMnO_3_. The activation and stability of lattice oxygen of reconstructed S-LaNiFeCoCrMnO_3_ for the OER achieve a balance, thus breaking the trade-off relationship between activity and stability for the LOM toward the OER. (iii) The increased CNs of Fe–O and Ni–O for S-LaNiFeCoCrMnO_3_ indicate that adsorption of the oxygen-containing intermediates was facilitated, which is an indication of favoring the enhancement of OER activity.

## Conclusions

We have synthesized a high-entropy S-LaNiFeCoCrMnO_3_ catalyst, and it exhibited excellent catalytic performance for the OER in an AEMWE. The existence of local Ni–S bonds in the sulfurized layer on the S-LaNiFeCoMnCrO_3_ surface facilitated *in situ* formation of the Fe–NiOOH active phase with a NiO_2_ subunit layer and high-valent Ni^4+^, enhancing the reactivity of lattice oxygen and improving the LOM for the OER. Such strong covalent Ni–O bonds and weak covalent Fe–O bonds in the Fe–NiOOH active phase play a crucial role in increasing the reactivity and stability of lattice oxygen, respectively, thus breaking the activity–stability trade-off and further improving the catalytic performance toward the OER. This work not only presents a highly active and stable catalyst for the OER at the anode in the AEMWE, but also deepens the understanding of the lattice oxygen mechanism to optimize the catalytic performance toward the OER.

## Author contributions

G. Wang and Z. Li conceived the project and revised the manuscript. X. Li, Z. Li, B. Chen, and M. Wang conducted the material synthesis and material characterization. C. Yan and Z. Li conducted the TEM and HRTEM measurements. X. Li and Z. Li conducted the electrochemical measurements. Z. Li, S. Jane, and Q. Li conducted the XANES measurement. Z. Li, X. Li, Z. Liao, and D. Gao completed the measurement using the anion exchange membrane water electrolyzer. All authors contributed to the manuscript.

## Conflicts of interest

The authors declare no competing financial interest.

## Supplementary Material

SC-016-D5SC04541J-s001

## Data Availability

The data supporting this article have been included as part of the SI. Supplementary information is available. See DOI: https://doi.org/10.1039/d5sc04541j.
