# Supplementary material for: Regulating metal–oxygen covalency in reconstructed sulfurized high-entropy perovskite to activate and stabilize lattice oxygen for the oxygen evolution reaction
Source: Chem Sci. 2025 Sep 17;16(42):19752–61. doi: 10.1039/d5sc04541j (PMC12486150; doi:10.1039/d5sc04541j)
Supplement: SC-016-D5SC04541J-s001 [file SC-016-D5SC04541J-s001.pdf]

Supplementary Information (SI) for Chemical Science.
This journal is © The Royal Society of Chemistry 2025

Supporting Information

Regulating metal-oxygen covalency in reconstructed sulfurized high-entropy perovskite to activate and stabilize lattice oxygen for oxygen evolution reaction

Xiang Li,[a,b] Qiuju Li,[c] Bingyu Chen,[a] Mengna Wang,[a,b] Chuanchuan Yan,[a,d] Subhajit Jana,[e] Ziqi Liao,[a,d] Zhenyu Li,[a,*] Dunfeng Gao,[a] Guoxiong Wang[a,*]

[a]State Key Laboratory of Catalysis Energy, Dalian National Laboratory for Clean Energy iChEM (Collaborative Innovation Center of Chemistry for Energy Materials), Dalian Institute of Chemical Physics Chinese Academy of Sciences, Dalian 116023, China
[b]Dalian Jiaotong University, Dalian 116028, China
[c]Department of Chemistry, College of Basic Medicine, Third Military Medical University (Army Medical University), Chongqing 400038, China
[d]University of Chinese Academy of Sciences, Beijing 100049, China
[e]Department of Mechanical and Mechatronics Engineering, Waterloo Institute for Nanotechnology, Materials Interfaces Foundry, University of Waterloo, Waterloo, Ontario N2L3G1, Canada

Corresponding authors
Email: lizhenyu@dicp.ac.cn (Z. Li); wanggx@dicp.ac.cn (G. Wang)

This file contains:
Experimental section
Figs. S1 to S38
Tables S1-S7
References (1-50)

1. Experimental Section

1.1 Chemicals and Reagents

$La(NO_3)_3 \cdot 6H_2O$, $Ni(NO_3)_2 \cdot 6H_2O$, $Fe(NO_3)_3 \cdot 9H_2O$, $Co(NO_3)_2 \cdot 6H_2O$, $Cr(NO_3)_3 \cdot 9H_2O$, and citric acid monohydrate ($C_6H_8O_7 \cdot H_2O$) were purchased from Macklin. KOH and ethanol were purchased from Beijing Chemical Factory. $Mn(NO_3)_2$ solution (50 wt% in H_2O) was obtained from Aladdin. The 5 wt% Nafion ionomer solution was purchased from Alfa Aesar. The commercial 70 wt% Pt/C catalyst was obtained from TANAKA. Highly purified water (>18 MΩ cm resistivity) was provided by PALL PURELAB Plus system.

1.2 Material Preparation

Synthesis of $LaNiFeCoCrMnO_3$. $La(NO_3)_3 \cdot 6H_2O$ (2.0 mmol), $Ni(NO_3)_2 \cdot 6H_2O$ (1.6 mmol), $Fe(NO_3)_3 \cdot 9H_2O$ (0.1 mmol), $Co(NO_3)_2 \cdot 6H_2O$ (0.1 mmol), $Cr(NO_3)_3 \cdot 9H_2O$ (0.1 mmol), and $Mn(NO_3)_2$ (0.1 mmol) were added into a beaker with 100 mL deionized water. Then, citric acid ($C_6H_8O_7 \cdot H_2O$) was added into the above solution under continuous ultrasound stirring for 10 min. Subsequently, the above mixed solution was heated at 200 °C for 2 h, until the liquid was evaporated completely and the obtained gray colloid was burned fully. The obtained gray powder was fully ground, transferred to a crucible, subsequently placed in a Muffle furnace, followed by heating to 850 °C at a heating rate of 1 °C/min and retained 850 °C for 6 h. After natural cooling, the $LaNiFeCoCrMnO_3$ catalyst was acquired.

Synthesis of S-$LaNiFeCoCrMnO_3$. The 100 mg of as-prepared $LaNiFeCoCrMnO_3$ sample and 500 mg of thiourea were placed in the upstream and downstream areas of the tube furnace, respectively. The tubular furnace is then heated to 350 °C at a heating rate of 3 °C/min in Ar atmosphere and remained at this temperature for 2 h. Finally, the resulting black sample for S-$LaNiFeCoCrMnO_3$ catalyst is obtained after washing several times with deionized water and ethanol.

Synthesis of $LaNiFeCoCrO_3$. The $LaNiFeCoCrO_3$ was synthesized by similar synthesis procedure as $LaNiFeCoCrMnO_3$ except without the addition of $Mn(NO_3)_2$.

Synthesis of $LaNiO_3$. The $LaNiO_3$ was synthesized by similar synthesis procedure as $LaNiFeCoCrMnO_3$ except without the addition of $Fe(NO_3)_3 \cdot 9H_2O$, $Co(NO_3)_2 \cdot 6H_2O$, $Cr(NO_3)_3 \cdot 9H_2O$, and $Mn(NO_3)_2$.

1.3 Catalyst Characterizations

The powder X-ray diffraction (XRD) patterns of the materials were conducted on X-ray diffractometer (Empyream) using Cu K_α radiation (λ = 1.5418 Å). Transmission electron microscopy (TEM) and high-resolution TEM (HRTEM) images were obtained by JEM-F200 microscope equipped with a field emission gun operating at 200 kV. X-ray photoelectron spectroscopy (XPS) spectra were recorded on a Thermofisher Escalab 250 Xi+ with photoelectron spectroscopy system using a monochromatic Al K_α (1486.6 eV). Aberration-corrected high-angle annular dark-field scanning transmission electron microscopy (HAADF-STEM) image was conducted on a JEOL-ARM 300F microscope at 300 kV. The scanning electron microscopy images of samples were obtained using a JSM-7800F with an accelerating voltage of 3 kV. The Raman spectra of samples were recorded using a NanoWizard equipment. The X-ray absorption spectra (XAS) including X-ray absorption near-edge structure (XANES) and extended X-ray absorption fine structure (EXAFS) of the samples at Fe K-edge (7112 eV) and Ni K-edge (8333 eV) were collected at the Singapore Synchrotron Light Source (SSLS) center, where a pair of channel-cut Si (111) crystals was used in the monochromator. The Fe K-edge XANES data were recorded in a transmission mode, and Fe foil FeO, and Fe_2O_3 were used as references. The Ni K-edge XANES data were recorded in a transmission mode, and Ni foil, NiO, and NiOOH were used as references.

1.4 Electrochemical Measurements in Three-Electrode System

All electrochemical tests were conducted with a CHI 660E electrochemical workstation. The glassy carbon electrode (GCE) with a diameter of 3 mm, Hg/HgO electrode, and Pt wire acted as the working electrode, reference electrode, and counter electrode, respectively. To prepare the catalyst-coated GCE, 8 mg of S-$LaNiFeCoCrMnO_3$ catalyst, 760 μL of isopropanol, and 40 μL of conductive polymer binder (5 wt% Nafion ionomer solution) were first mixed and ultrasonicated for 30 min to form the uniform catalyst ink. Then, 2 μL of the catalyst ink was dropped onto the GCE and then dried naturally. The loading amount of S-$LaNiFeCoCrMnO_3$ catalyst was 0.281 mg/cm^2 on the surface of GCE. The potential of zero net current for Hg/HgO electrode was calibrated to be 0.098 V by using a reversible hydrogen electrode (RHE) before testing. Thus, the potentials, measured against Hg/HgO electrode, were converted into the potentials versus RHE by using the equation:

$$E_{\mathrm{vs.RHE}} = E_{\mathrm{vs.Hg/HgO}} + 0.098\ \mathrm{V} + 0.059\mathrm{pH} \quad (1)$$

Linear sweep voltammetry (LSV) measurements were conducted in 1.0 M KOH within the potential range of 0.9 V to 1.7 V *vs*. RHE at the scan rate of 1 mV/s and compensated by 85% *iR*-

drop. The chronopotentiometric curve of S-$LaNiFeCoCrMnO_3$ was measured at the current density of 10 mA/cm^2.

The electrochemical active surface area (ECSA) of different catalysts was obtained by measuring the CVs with different scan rates (10, 20, 30, 50, 70, 90, and 100 mV/s) under the potential range between 0.80 and 1.15 V *vs*. RHE. To ensure the consistency of CVs, the sweep segments were set to 60 segments. Subsequently, the linear relationship between the difference of current density $\Delta J = (J_{anodic} - J_{cathodic})/2$ at 0.975 V *vs*. RHE against the scan rate was plotted, which then the geometric double layer capacitance (C_{dl}) was obtained by calculating the slope of the linear trend. Finally, the ECSAs of catalysts on GCE were calculated by the following equation 2:

$$ESCA = \frac{C_{dl}}{C_s} \quad (2)$$

where C_s is the specific capacitance of the sample, and the value of C_s is 0.06 mF/cm^2.

The Faradaic efficiency (FE) of S-$LaNiFeCoCrMnO_3$ catalyst was estimated by the drainage water method, where the practical amount of generated O_2 during the OER was known by using the ideal gas law. The theoretical amount of generated O_2 was calculated by using the Faraday law, which build on the basics of the assumption that the working electrode only occurred the OER. The ratio between the practical amount of generated O_2 and the theoretical amount of generated O_2 is the Faradaic efficiency of a catalyst for the OER. The FE is calculated by following equation 3:

$$FE = \frac{Practical\ amount\ of\ generated\ O_2}{Theoretical\ amount\ of\ generated\ O_2} \quad (3)$$

1.5 *In situ* Raman Spectra Measurements

Coupling both a NanoWizard equipment and a CHI 660E electrochemistry workstation to conduct the measurement of in situ Raman spectra of catalysts during the OER. The Raman was set with a 20 mW air-cooled argon-ion laser (532 nm) as the excitation light source while operating at different overpotentials (0 mV, 100 mV, 200 mV, 300 mV, 400 mV, and 500 mV) toward the OER, respectively.

1.6 *On line* DEMS Measurements

The participation ratio of lattice oxygen mechanism was measured using *on line* differential electrochemical mass spectrometry (DEMS) provided by Ling Lu Instruments (Shanghai) Co., LTD coupled electrochemical workstation. The membrane was provided by Ling Lu Instruments

(Shanghai) Co., LTD, and the membrane with pore size of ≤ 20 nm, and pore distribution of ≥ 50% was used in *on line* DEMS experiment. For *on line* differential electrochemical mass spectrometry measurements, we recorded the LSV curves when the ^{18}O isotope-labeling catalysts used to catalyze the OER in 1.0 M KOH with $H_2{}^{16}O$. (i) To obtain the ^{18}O isotope-labeling S-$LaNiFeCoCrMnO_3$ working electrode, the S-$LaNiFeCoCrMnO_3$ working electrode was subjected to chronopotentiometric curve at 1.5 V *vs* RHE in 1.0 M KOH solution with $H_2{}^{18}O$ as solvent; (ii) To remove the residual $H_2{}^{18}O$ on the surface for the above ^{18}O isotope-labeling S-$LaNiFeCoCrMnO_3$ electrode, $H_2{}^{16}O$ was used as solvent to wash the electrode surface and subsequently several CV measurements were conducted within the potential from 0.6 V to 1.2 V *vs*. RHE at 50 mV/s. (iii) The LSV cycles of ^{18}O isotope-labeling S-$LaNiFeCoCrMnO_3$ electrode were measured in 1.0 M KOH solution with $H_2{}^{16}O$ as electrolyte within the potential range of 1.1~1.7 V *vs*. RHE, which then the gaseous products of $^{36}O_2$, $^{34}O_2$, and $^{32}O_2$ were monitored by the mass spectrometer. The ratio of $^{36}O_2$, $^{34}O_2$ and $^{32}O_2$ can be estimated by calculating the integral areas for the corresponding mass signals.

1.7 Electrochemical Measurements in AEMWE

In order to construct the membrane electrode assembly (MEA), the S-$LaNiFeCoCrMnO_3$ was used as anode catalyst, while the commercial 70 wt% Pt/C catalyst was used as cathode catalyst. In a typical experiment, S-$LaNiFeCoCrMnO_3$ and Pt/C were added into the mixed solution of water and isopropanol with a ratio of 1:1, respectively. The Nafion ionomer solution (5 wt%) was added into the S-$LaNiFeCoCrMnO_3$-containing ink and Pt/C-containing ink. The ratio of the mass of Nafion ionomer to the mass of S-$LaNiFeCoCrMnO_3$ catalyst is 15 wt%, while the ratio of the mass of Nafion ionomer to the mass of Pt/C catalyst is 30 wt%. Subsequently, the above two solution were ultrasonicated for 1 h under ice-bath condition to obtain the uniform catalyst inks. Subsequently, the S-$LaNiFeCoCrMnO_3$ with a loading amount of 2 mg/cm^2 and commercial Pt/C with a Pt loading amount of 0.5 mg/cm^2 were sprayed on Ti felt and carbon paper, respectively. Ti felt and carbon paper were acted as anode gas diffusion layer and cathode gas diffusion layer, respectively. S-$LaNiFeCoCrMnO_3$-coated Ti felt and commercial Pt/C-coated carbon paper were pressed on the both sides of alkaline exchange membrane (AEM) to construct the MEA. After the MEA was constructed, the polarization curves of AEMWE with S-$LaNiFeCoCrMnO_3$ anode catalyst were obtained by circulating 1.0 M KOH while remaining the cell temperature at 80 ºC. The stability test of anion exchange membrane

water electrolyzer with S-$LaNiFeCoCrMnO_3$ anode catalyst was evaluated by recording the chronopotentiometric curve at 1.0 A/cm^2.

2. Theoretical Calculations

Spin-polarized DFT calculations were performed by the Vienna ab initio simulation package (VASP).[1] The core-electrons and exchange-correlation energy were treated with the projector-augmented-wave (PAW) method and Perdew−Burke−Ernzerhof (PBE) functional within the generalized gradient approximation (GGA), respectively.[2-3] A cutoff energy of 400 eV along with a 2 × 2 × 1 Monkhorst−Pack k-points grid was adopted for geometry relaxation. The convergence thresholds for energy and force were set as 10^{-5} eV and 0.05 eV/Å, and a vacuum space of 15 Å was set along the c direction. Additionally, the DFT-D3 empirical dispersion correction was used to describe the long-range van der Waals interactions.[4] The γ-NiOOH and Fe-doped γ-NiOOH were used to model the catalysts along with the DFT+U method.[5] The structures and U values were determined according to the work of Friebel et al., with values of 6.6 eV for Ni and 3.5 eV for Fe, respectively.[6] After the structural optimization was completed, we calculated the electron localization function (ELF) within a self-consistent framework. A 9×9×1 k-point grid was adopted to calculate Density of states (DOS).[7] The crystal orbital Hamilton population (COHP) was performed by LOBSTER.[8]

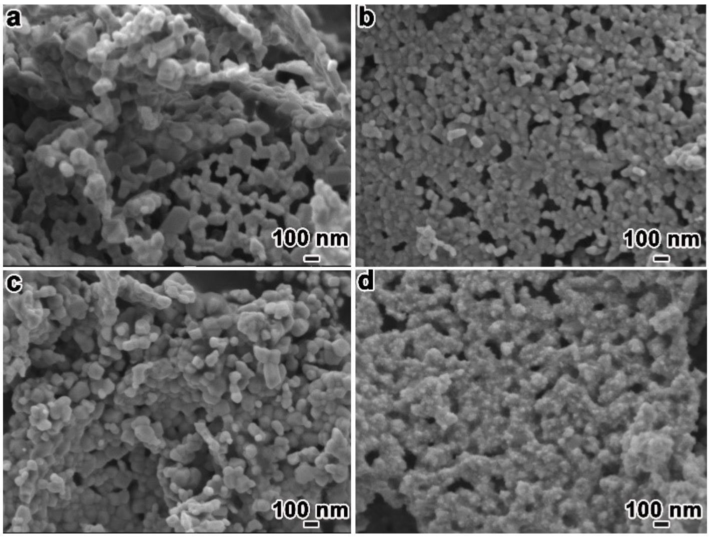

Figure S1. (a-d) SEM images of $LaNiO_3$, $LaNiFeCoCrO_3$, $LaNiFeCoCrMnO_3$, and S-$LaNiFeCoCrMnO_3$ catalysts.

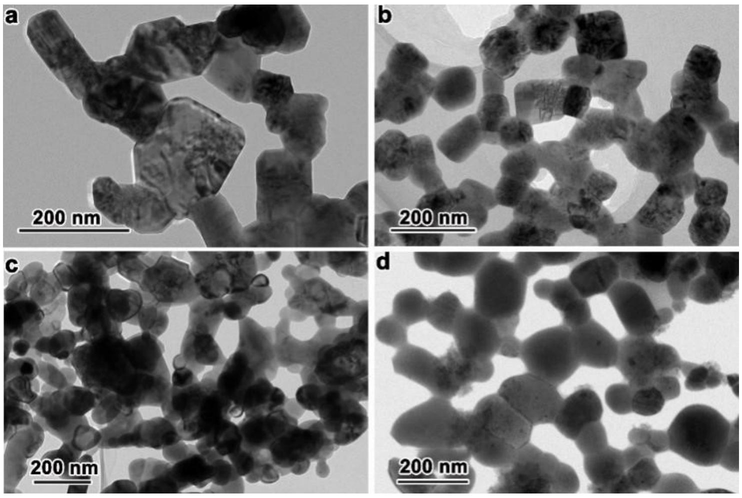

Figure S2. (a-d) TEM images of $LaNiO_3$, $LaNiFeCoCrO_3$, $LaNiFeCoCrMnO_3$, and S-$LaNiFeCoCrMnO_3$ catalysts.

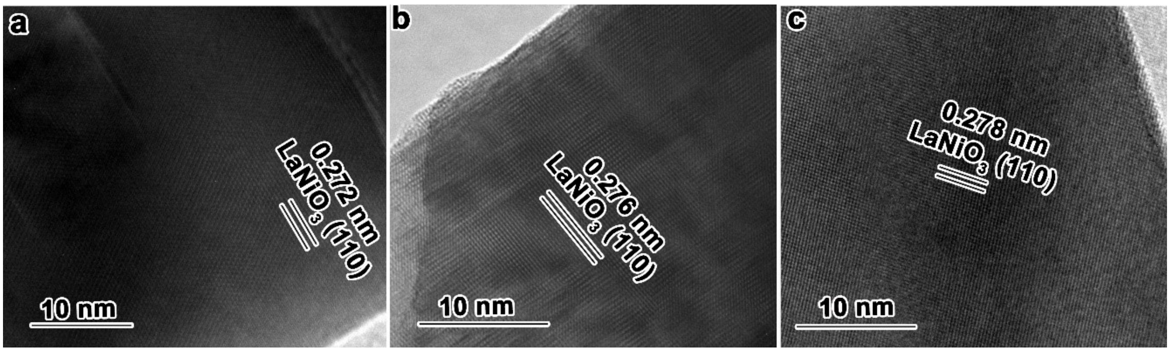

Figure S3. (a-c) HRTEM images of $LaNiO_3$, $LaNiFeCoCrO_3$, and $LaNiFeCoCrMnO_3$ catalysts.

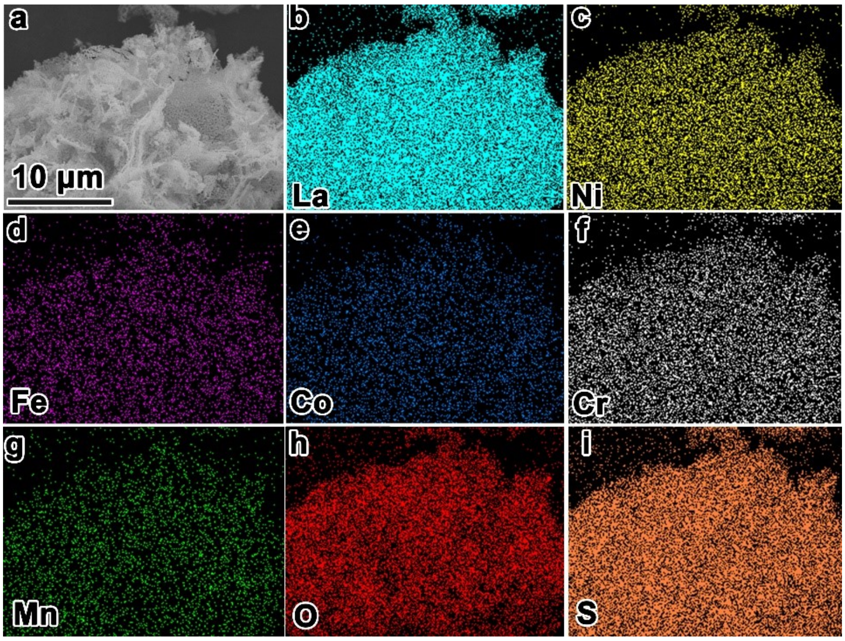

Figure S4. SEM image (a) and elemental maps (b-i) of S-$LaNiFeCoCrMnO_3$ catalyst.

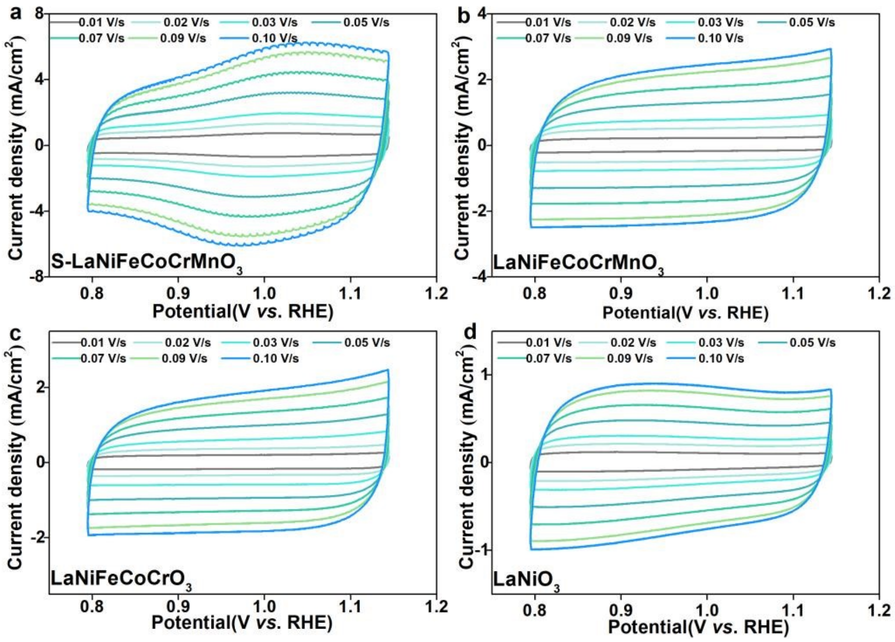

Figure S5. (a-d) Cyclic voltammograms of $LaNiO_3$, $LaNiFeCoCrO_3$, $LaNiFeCoCrMnO_3$, and S-$LaNiFeCoCrMnO_3$ catalysts in the potential region of 0.8~1.15 V *vs*. RHE at various scan rates.

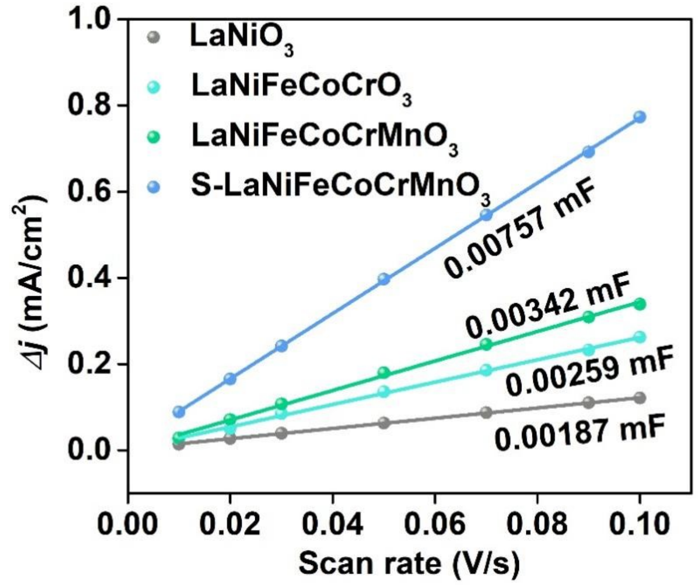

Figure S6. The double-layer capacitance (C_{dl}) of $LaNiO_3$, $LaNiFeCoCrO_3$, $LaNiFeCoCrMnO_3$, and S-$LaNiFeCoCrMnO_3$ catalysts by fitting their slopes of current between the anodic and cathodic sweeps versus scan rate.

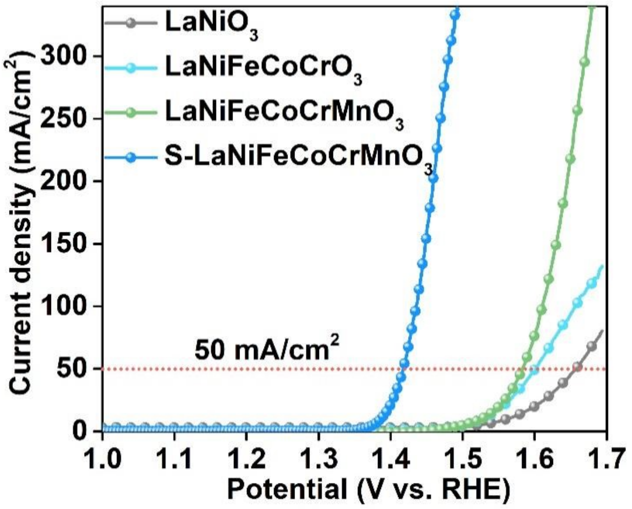

Figure S7. LSV curves of $LaNiO_3$, $LaNiFeCoCrO_3$, $LaNiFeCoCrMnO_3$, and S-$LaNiFeCoCrMnO_3$ catalysts by normalizing current to ECSA.

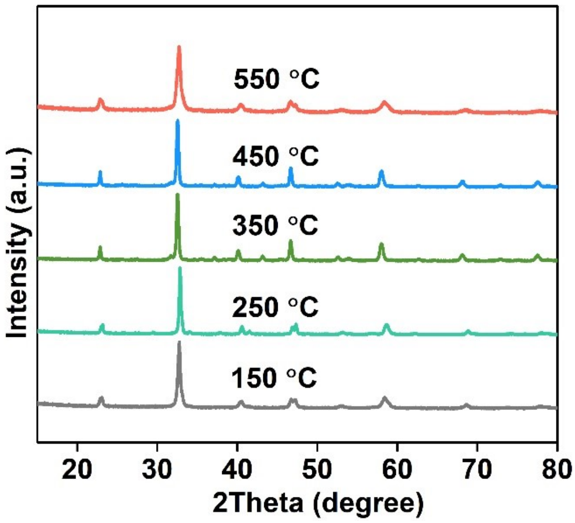

Figure S8. XRD patterns of S-$LaNiFeCoCrMnO_3$ catalysts prepared at different temperatures.

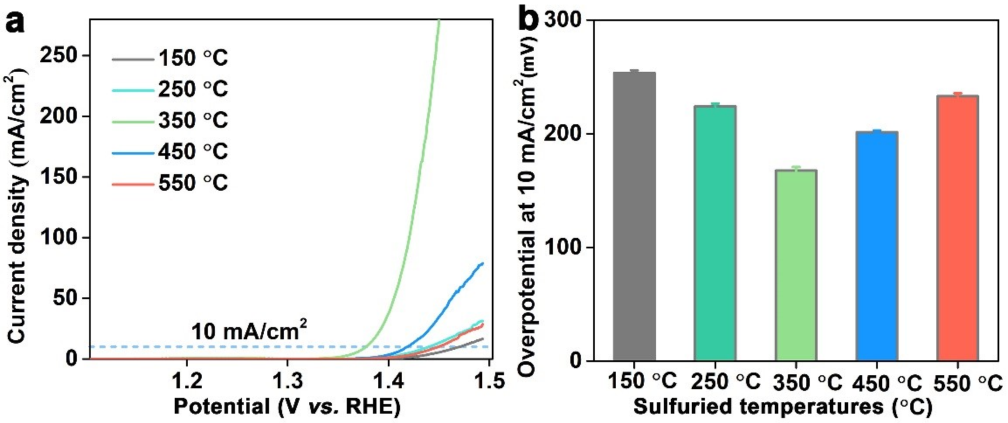

Figure S9. LSV curves and the corresponding overpotential comparison at 10 mA/cm^2 for S-LaNiFeCoCrMnO$_3$ catalysts prepared at different temperatures.

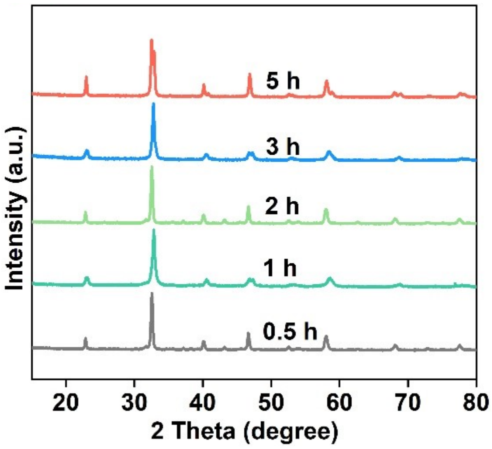

Figure S10. XRD patterns of S-$LaNiFeCoCrMnO_3$ catalysts prepared at different sulfuration time.

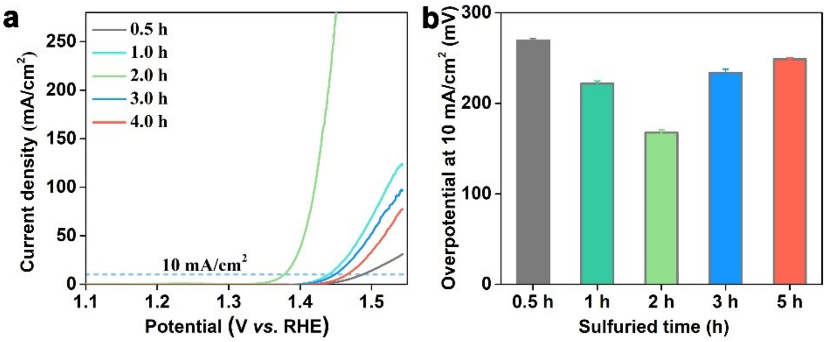

Figure S11. (a-b) LSV curves and the corresponding overpotential comparison at 10 mA/cm^2 for S-LaNiFeCoCrMnO$_3$ catalysts prepared at different sulfuration time.

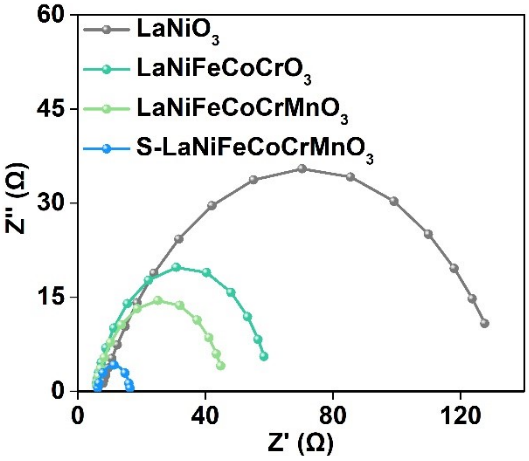

Figure S12. EIS spectra of $LaNiO_3$, $LaNiFeCoCrO_3$, $LaNiFeCoCrMnO_3$, and S-$LaNiFeCoCrMnO_3$ catalysts for the OER.

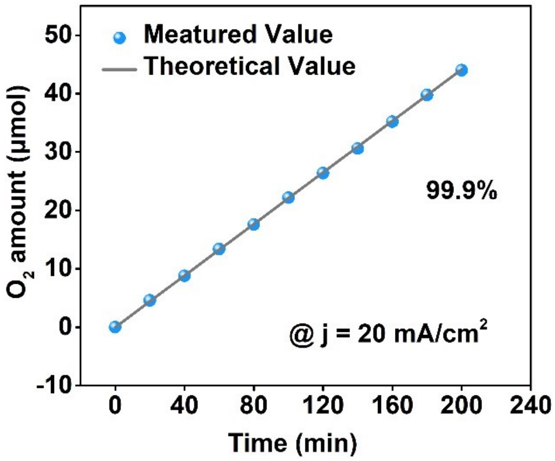

Figure S13. Faradaic efficiency of O_2 over S-$LaNiFeCoCrMnO_3$ catalyst at 20 mA/cm^2 toward the OER.

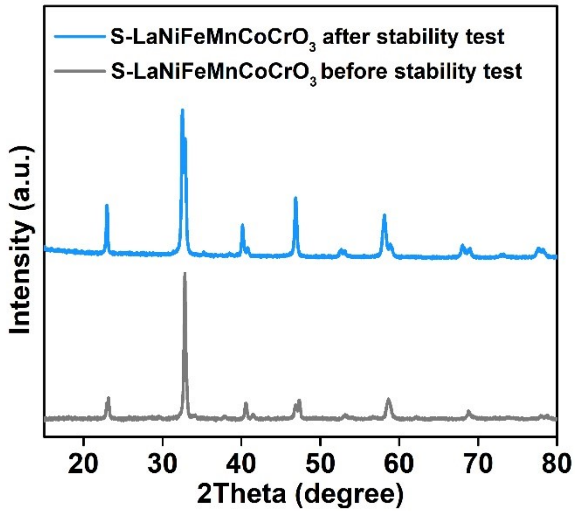

Figure S14. XRD patterns of S-$LaNiFeCoCrMnO_3$ catalysts before and after stability test toward the OER.

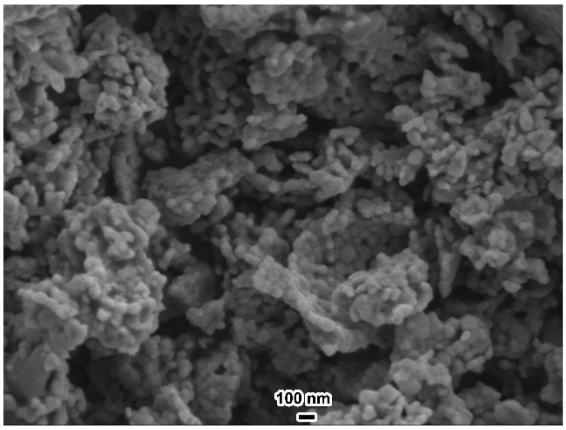

Figure S15. SEM image of S-$LaNiFeCoCrMnO_3$ catalyst after stability test toward the OER.

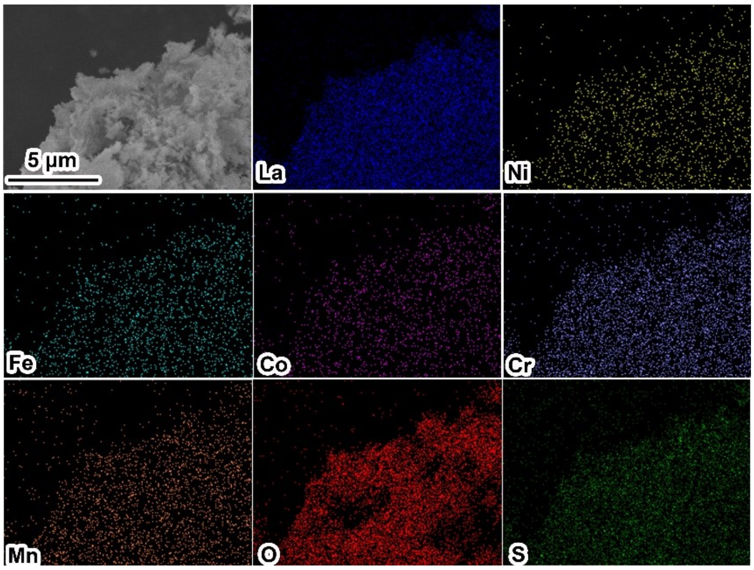

Figure S16. SEM image and elemental maps of S-$LaNiFeCoCrMnO_3$ catalyst after stability test toward the OER.

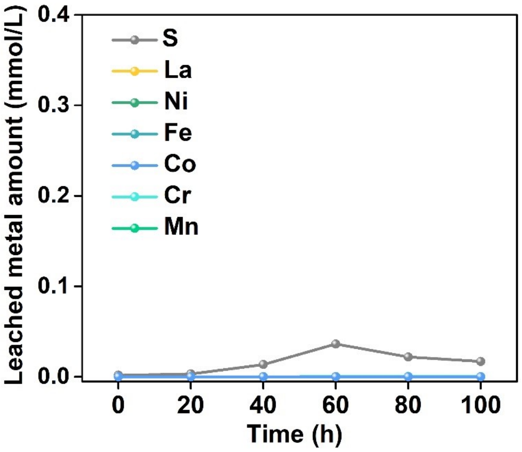

Figure S17. Leached amount of La, Ni, Fe, Co, Cr, Mn, and S elements for S-$LaNiFeCoCrMnO_3$ catalyst during OER.

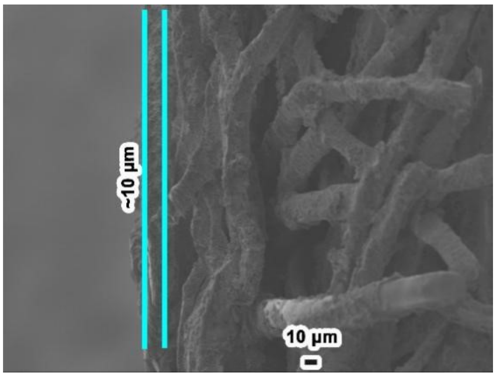

Figure S18. Cross-sectional SEM image of S-$LaNiFeCoCrMnO_3$-coated membrane before stability test in AEMWE.

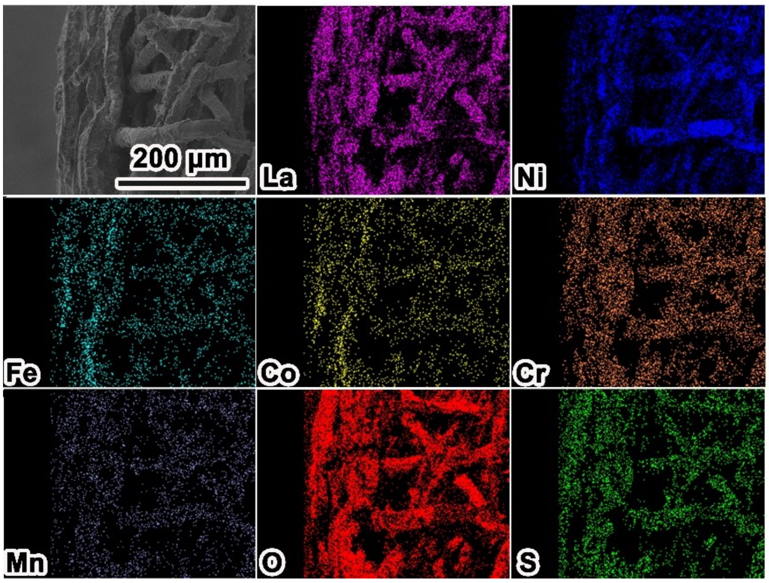

Figure S19. Cross-sectional SEM image and elemental maps of S-$LaNiFeCoCrMnO_3$-coated membrane before stability test in AEMWE.

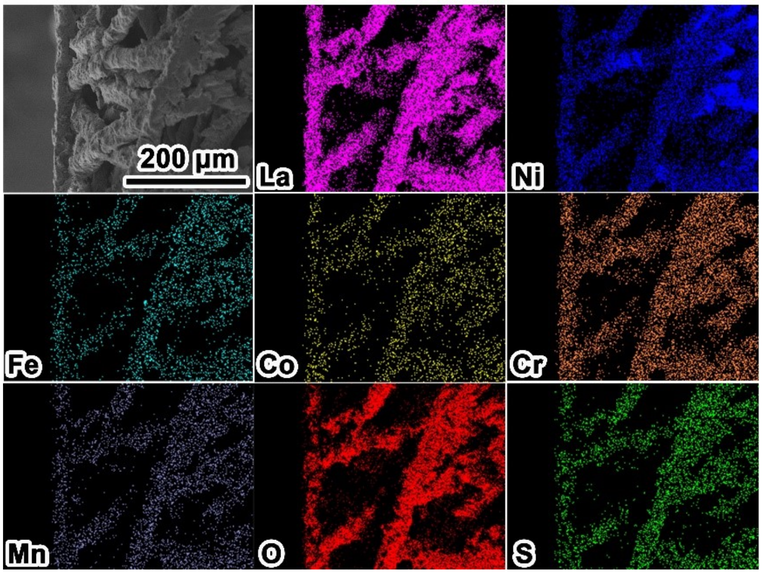

Figure S20. SEM image and elemental maps of S-$LaNiFeCoCrMnO_3$-coated membrane after stability test in AEMWE.

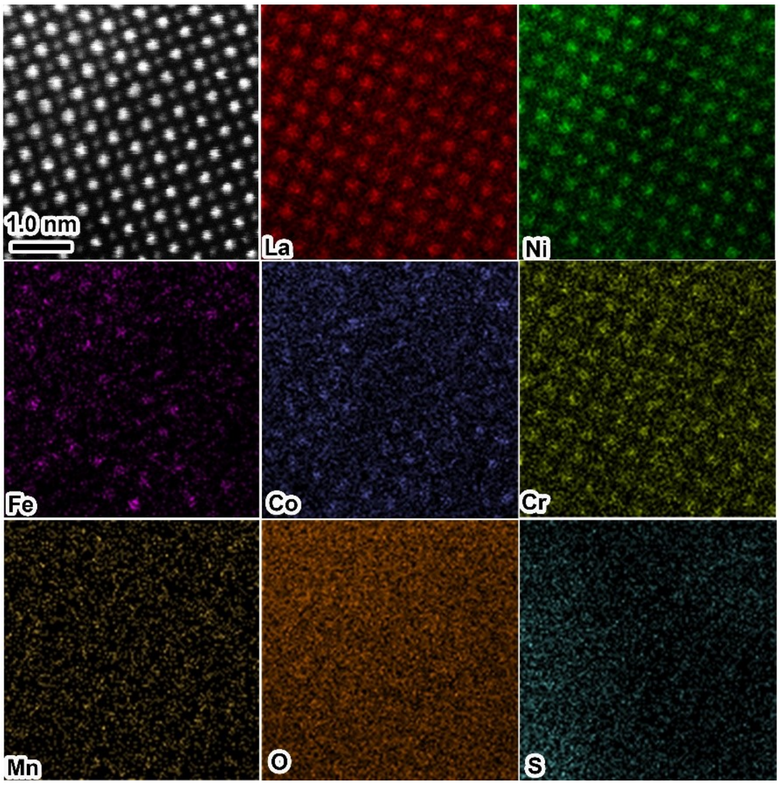

Figure S21. Enlarged HAADF-STEM image and elemental maps of La, Ni, Fe. Co, Cr, Mn, O, and S in S-LaNiFeCoCrMnO$_3$ catalyst after stability test in three-electrode system.

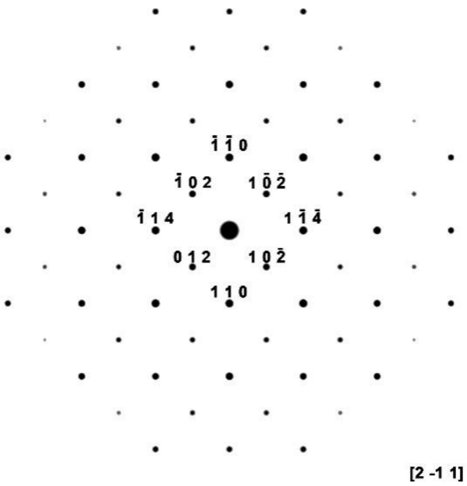

Figure S22. Simulated electron diffraction pattern of S-$LaNiFeCoCrMnO_3$ catalyst along with the [2 -1 1] direction.

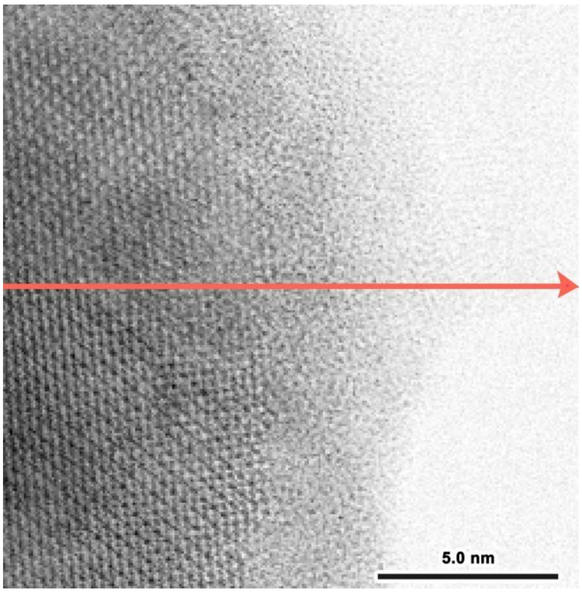

Figure S23. Atom-level line scanning region for EDX spectra in HAADF-STEM image of S-$LaNiFeCoCrMnO_3$ catalyst.

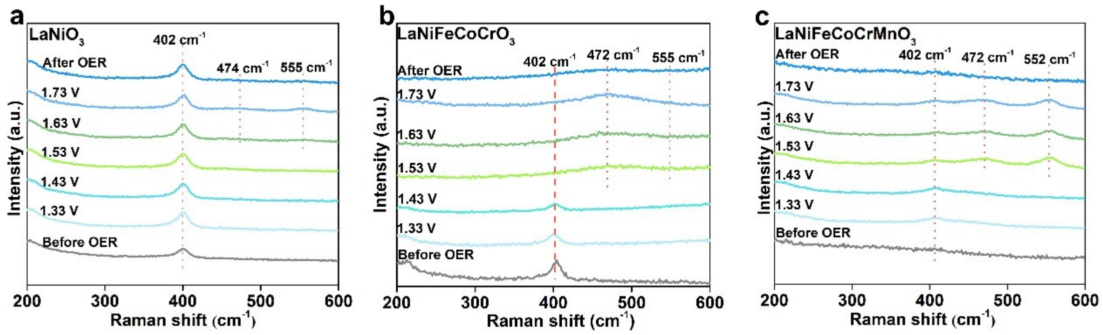

Figure S24. (a-c) *In situ* Raman spectra of $LaNiO_3$, $LaNiFeCoCrO_3$, and $LaNiFeCoCrMnO_3$ catalysts at different stages during the OER.

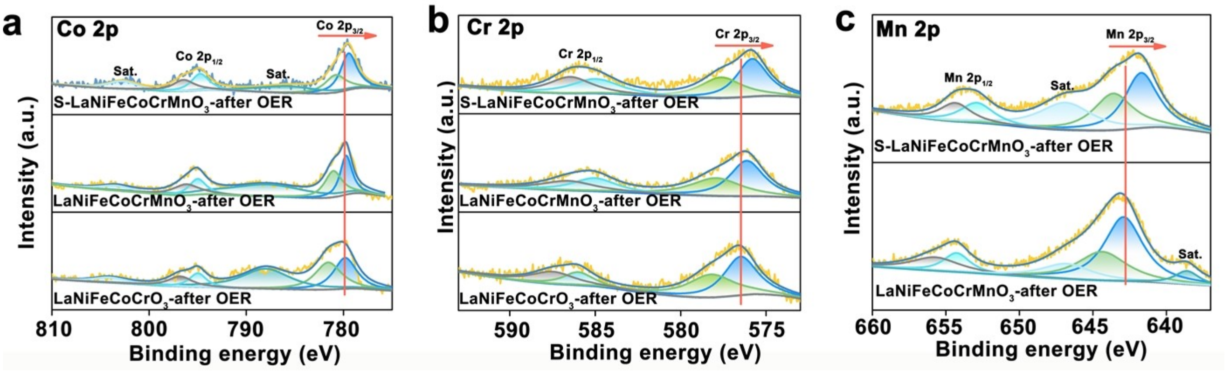

Figure S25. (a) Co 2p XPS spectra of $LaNiFeCoCrO_3$, $LaNiFeCoCrMnO_3$, and S-$LaNiFeCoCrMnO_3$ catalysts after stability test. (b) Cr 2p XPS spectra of $LaNiFeCoCrO_3$, $LaNiFeCoCrMnO_3$, and S-$LaNiFeCoCrMnO_3$ catalysts after stability test. (c) Mn 2p XPS spectra of $LaNiFeCoCrMnO_3$ and S-$LaNiFeCoCrMnO_3$ catalysts after stability test.

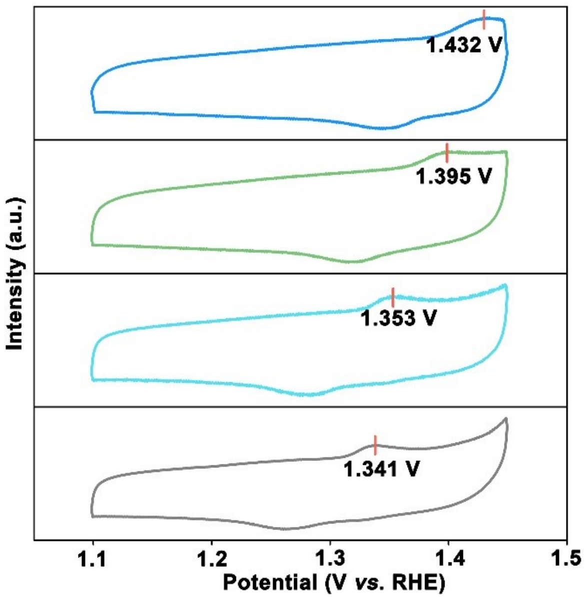

Figure S26. CVs of $LaNiO_3$, $LaNiFeCoCrO_3$, $LaNiFeCoCrMnO_3$, and S-$LaNiFeCoCrMnO_3$ catalysts.

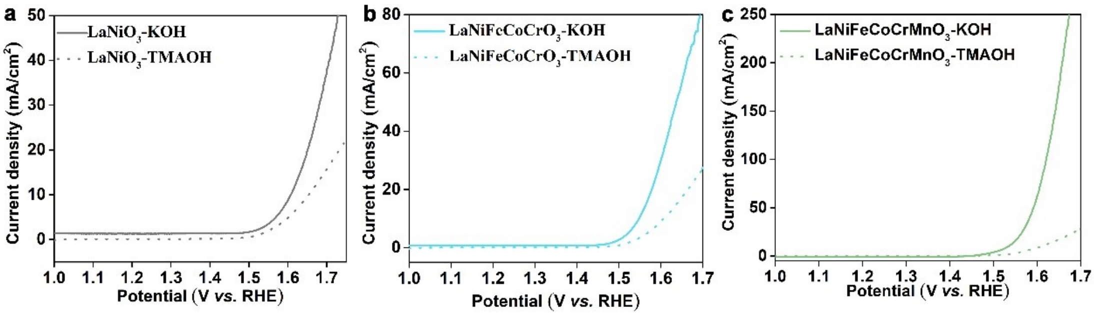

Figure S27. (a-c) LSV curves of $LaNiO_3$, $LaNiFeCoCrO_3$, and $LaNiFeCoCrMnO_3$ catalysts in 1.0 M KOH and 1.0 M TMAOH.

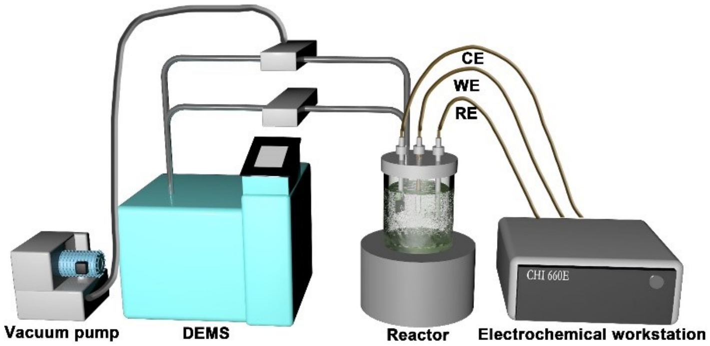

Figure S28. Schematic illustration of *on line* DEMS during OER. WE, CE, and RE represent the working electrode, counter electrode, and reference electrode, respectively.

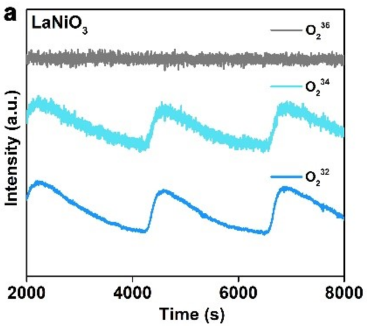

Figure S29. DEMS signals of O_2 products for ^{18}O-labeled $LaNiO_3$, $LaNiFeCoCrO_3$, and $LaNiFeCoCrMnO_3$ catalysts in 1.0 M KOH with $H_2{}^{16}O$.

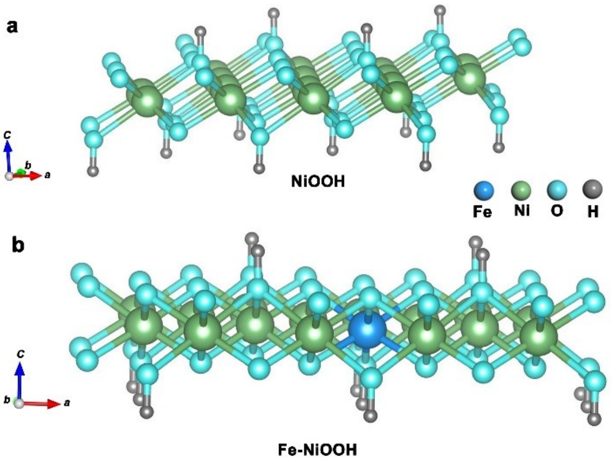

Figure S30. Theoretical models of NiOOH and Fe-NiOOH.

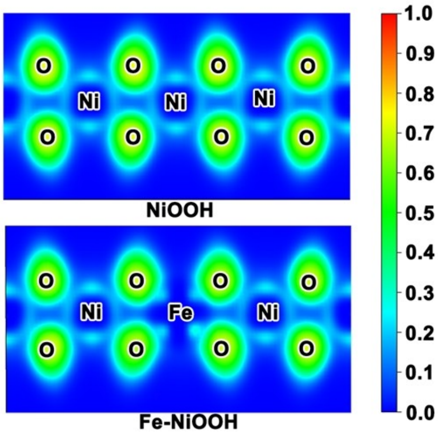

Figure S31. ELF maps of NiOOH and Fe-NiOOH.

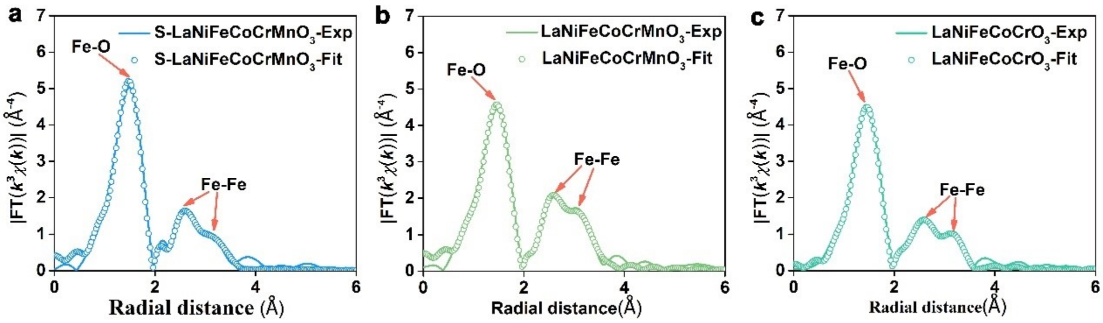

Figure S32. (a-c) Fitting data of Fourier-transformed XANES of Fe k-edge for $LaNiFeCoCrO_3$, $LaNiFeCoCrMnO_3$, and S-$LaNiFeCoCrMnO_3$ catalysts after stability test toward the OER.

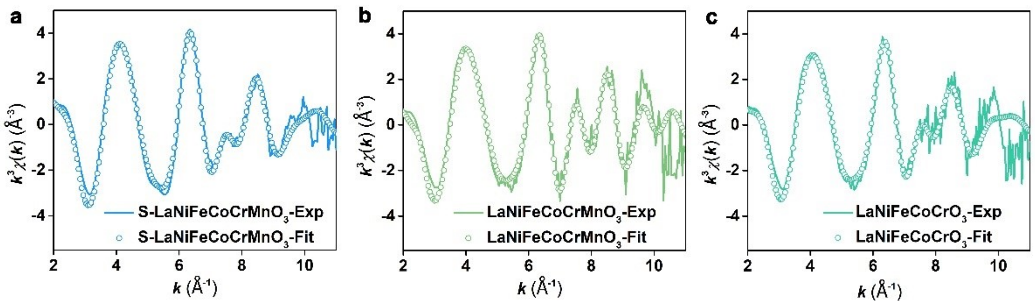

Figure S33. (a-c) Fourier transformed k space and the corresponding fitted curves of Fe k-edge for $LaNiFeCoCrO_3$, $LaNiFeCoCrMnO_3$, and S-$LaNiFeCoCrMnO_3$ catalysts after stability test toward the OER.

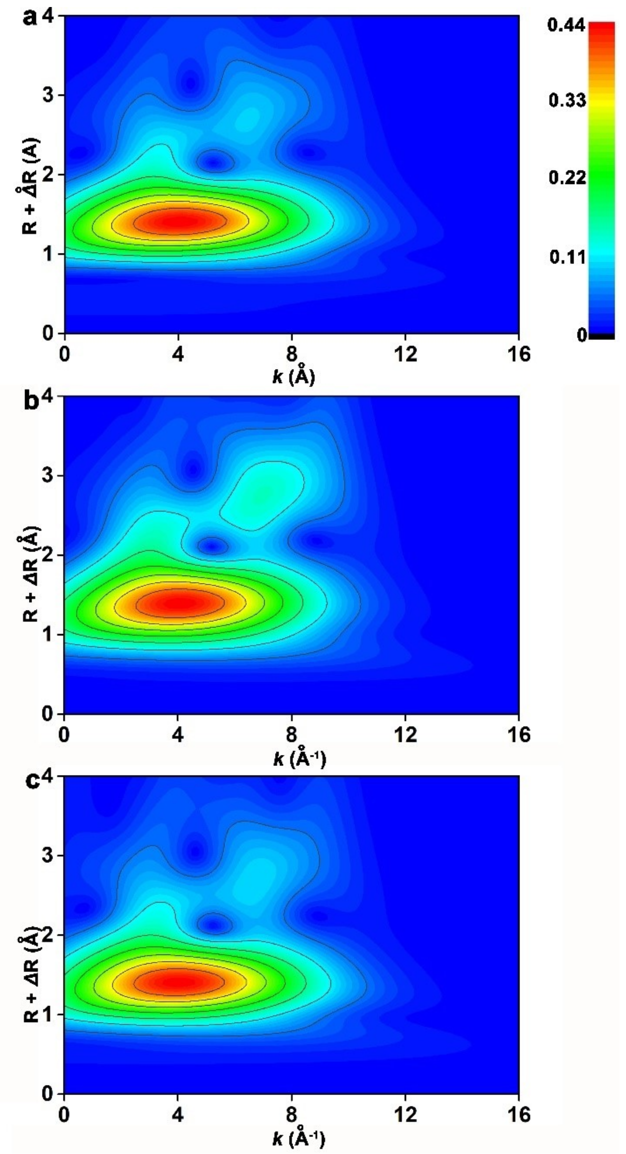

Figure S34. (a-c) WT-EXAFS of Fe k-edge for $LaNiFeCoCrO_3$, $LaNiFeCoCrMnO_3$, and S-$LaNiFeCoCrMnO_3$ catalysts after stability test toward the OER.

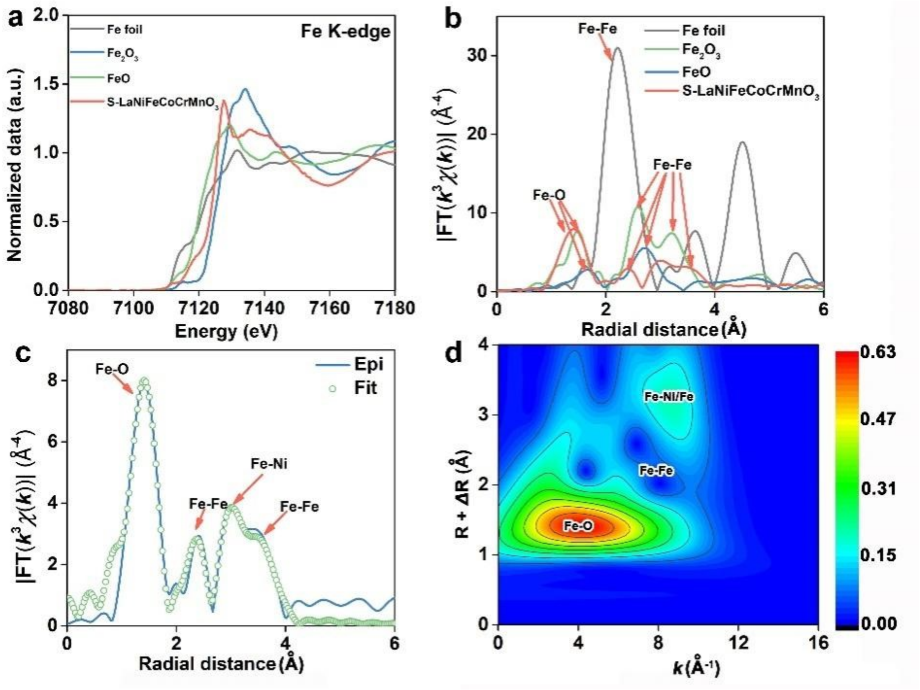

Figure S35. (a-d) XANES, Fourier-transformed EXAFS, fitting data of Fourier-transformed XANES, and WT-EXAFS of Fe K-edge for S-LaNiFeCoCrMnO$_3$ catalyst before the OER.

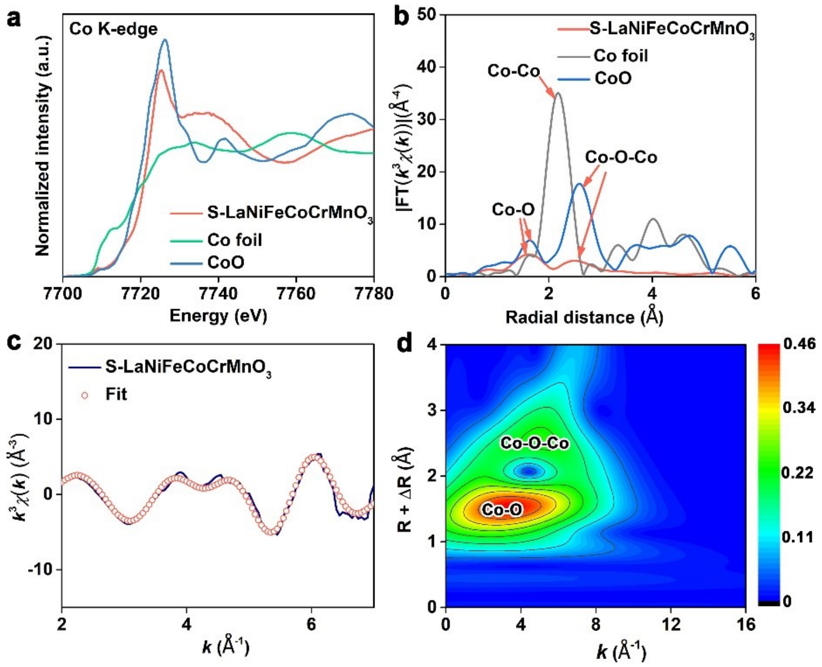

Figure S36. (a-d) XANES, Fourier-transformed EXAFS, fitting data of Fourier-transformed XANES, and WT-EXAFS of Co K-edge for S-$LaNiFeCoCrMnO_3$ catalyst before the OER.

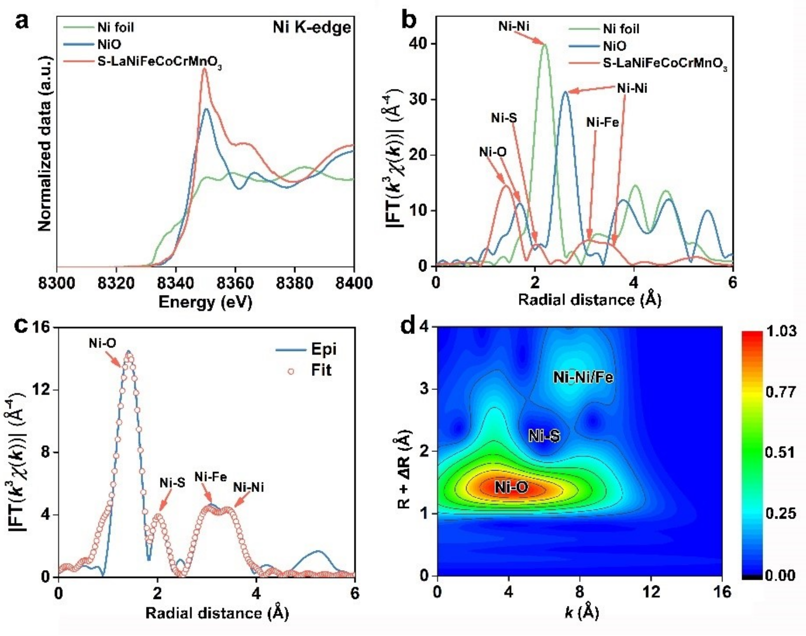

Figure S37. (a-d) XANES, Fourier-transformed EXAFS, fitting data of Fourier-transformed XANES, and WT-EXAFS of Ni K-edge for S-$LaNiFeCoCrMnO_3$ catalyst before the OER.

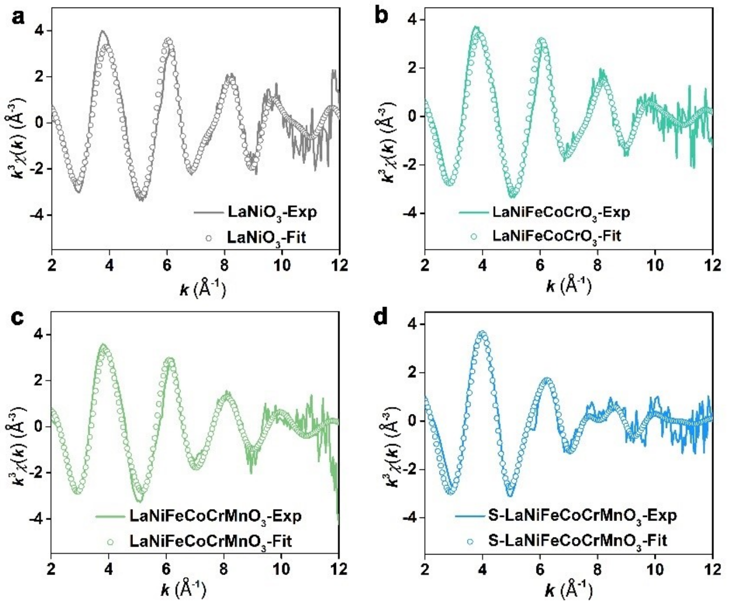

Figure S38. (a-d) Fourier transformed k space and the corresponding fitted curves of Ni k-edge for $LaNiO_3$, $LaNiFeCoCrO_3$, $LaNiFeCoCrMnO_3$, and S-$LaNiFeCoCrMnO_3$ catalysts after stability test toward the OER.

Table S1. Comparison of OER performance of S-$LaNiFeCoCrMnO_3$ with some representative catalysts in three-electrode system with alkaline media.

Catalysts	Overpotential at 10 mA/cm^2 (mV)	Stability (h)	Electrolyte	References
**S-$LaNiFeMnCoCrO_3$**	**165 mV**	**1800 h**	**1.0 M KOH**	**This work**
$FeNiCoCrMnS_2$	199 mV	55 h	1.0 M KOH	[9]
$(FeCoNiB_{0.75})_{97}Pt_3$	170 mV	200 h	1.0 M KOH	[10]
$(BSCF)_{3/4}[KM(II)F_3]_{1/4}$	345 mV	20 h	1.0 M KOH	[11]
$Mo(NiFeCo)_4/Ni$	200 mV	500 h	1.0 M KOH	[12]
$PrBa_{0.5}Ca_{0.5}Co_2O_{5+\delta}$	300 mV	0.2 h	1.0 M KOH	[13]
$(NiCo)S_{1.33}$	302 mV	10 h	1.0 M KOH	[14]
$Ba_{0.5}Sr_{0.5}Co_{0.8}Fe_{0.2}O_{3-\delta}$	387 mV	6 h	1.0 M KOH	[15]
p-SnNiFe	350 mV	6 h	1.0 M KOH	[16]
$Co_9S_8/Co_3S_4/Cu_2S$	181 mV	1.5 h	1.0 M KOH	[17]
Fe-NiO/NiS_2	270 mV	10 h	1.0 M KOH	[18]
$LaFe_xNi_{1-x}O_3$	302 mV	20 h	1.0 M KOH	[19]
CoMoNiS-NF-31	225 mV	20 h	1.0 M KOH	[20]
$K(MgMnFeCoNi)F_3$	369 mV	15 h	1.0 M KOH	[21]
$MAPbBr_3$@AlPO-5	233 mV	32 h	1.0 M KOH	[22]
$FeCoNiS_x$	202 mV	500 h	1.0 M KOH	[23]
CeO_2-$CoS_{1.97}$	189 mV	100 h	1.0 M KOH	[24]
F-CoO NNAs	213 mV	300 h	1.0 M KOH	[25]
G-FeCoW	191 mV	500 h	1.0 M KOH	[26]
$CuCo_2O_{4-x}S_x$/NC-2	241 mV	300 h	1.0 M KOH	[27]
$(CrFeCoNiMo)_3O_4$	255 mV	200 h	1.0 M KOH	[28]
Sm−$LaCoO_3$	530 mV	50 h	1.0 M KOH	[29]
$Bi_5CoTi_3O_{15}$	320 mV	90 h	1.0 M KOH	[30]

Table S2. Comparison of OER performance of S-$LaNiFeCoCrMnO_3$ with some representative catalysts in AEMWE.

Catalysts	Voltage@Current Density (V@A/cm^2)	Stability (h@A/cm^2)	Electrolyte	References
**S-$LaNiFeMnCoCrO_3$**	**2.0 V@5.8 A/cm^2 1.61 V@1.0 A/cm^2**	**140 h@1.0 A/cm^2**	**1.0 M KOH**	**This work**
$CoFe_2S_4$	-	72 h@0.2 A/cm^2	1.0 M KOH	[31]
$NiFe_{0.25}C_{r1.75}O_4$	1.64 V@1.0 A/cm^2	85 h@0.2 A/cm^2	1.0 M KOH	[32]
$CoCrO_x$	2.1 V@1.5 A/cm^2	120 h@0.5 A/cm^2	0.5 M KOH	[33]
(Ni, Fe)$_3S_2$/NFF	1.93 V@0.6 A/cm^2	420 h@0.6 A/cm^2	1.0 KOH	[34]
Ru–BO_x–OH-300	1.95 V@1.0 A/cm^2	200 h@1.0 A/cm^2	1.0 M KOH + seawater	[35]
NiCo@NiFe-LDH	2.0 V@3.61 A/cm^2	700 h@1.0 A/cm^2	1.0 M KOH	[36]
VCoP-0.1	2.0 V@3.1 A/cm^2	200 h@1.0 A/cm^2	1.0 M KOH	[37]
NiFe-BTC-GNPs MOF	1.85 V @1.15 A/cm^2	72 h@0.45 A/cm^2	0.1 M KOH	[38]
WC_{1-x}/Mo_2C@CNNCF	1.82 V@0.1 mA/cm^2	100 h@0.2 mA/cm^2	1.0 M KOH	[39]
$Ni_{78}Fe_{22-P}$ nanochain arrays	1.75 V@1.0 A/cm^2	210 h@0.1 A/cm^2	1.0 M KOH	[40]
RuZn-Co_3O_4	-	100 h@0.5 A/cm^2	1.0 M KOH	[41]
HTS-Ru-NCs/TiN	1.65 V@1.0 A/cm^2	160 h@0.5 A/cm^2	1.0 M KOH	[42]
lamellar fern-like alloy aeroge	1.65 V@1.0 A/cm^2.	1300 h@2.0 A/cm^2	6.0 M KOH.	[43]
d-(Fe,Ni)OOH	1.795 V@0.5 A/cm^2	96 h@2.0 A/cm^2	1.0 M KOH	[44]
(NiCo)$_3Se_4$(NiCoOOH)	2.0 V@2.0 A/cm^2	95 h@2.0 A/cm^2	1.0 M KOH	[44]
Nickel–iron-based electrocatalyst	2.0 V@7.35 A/cm^2	1800 h at 1.0 A/cm^2	1.0 M KOH	[45]
Fe-Ni_xO_y/Ni foam	2.3 V@10 A/cm^2	800 h at 10 A/cm^2	1.0 M KOH	[46]
$NiCo_2O_4$@FeNi LDH nanowire arrays	2 V@3.61 A/cm^2	700 h at 1 A/cm^2	1.0 M KOH	[36]
NiFe	2.0 V@3.0 A/cm^2	430 h at 1.6 A cm^2	6.0 M KOH	[47]
NiFe-LDH/NF	2.0 V@7.03 A/cm^2	250 h at 2 A/cm^2	1.0 M KOH	[48]
NiFe-LDH	2.0 V@ 5 A/cm^2	100 h at 1 A/cm^2	1.0 M KOH	[49]
$NiMoO_4 \cdot xH_2O$	2.0 V@6.14 A/cm^2	230 h at 1 A/cm^2	1.0 M KOH	[50]

Table S3. EXAFS data fitting results of Fe K-edge for Fe foil, FeOOH reference, FeO reference, Fe_2O_3 reference, $LaNiFeCoCrO_3$, $LaNiFeCoCrMnO_3$, and S-$LaNiFeCoCrMnO_3$ catalysts after stability test for the OER.

**Samples**	**Path**	***CN***[a]	***R*(Å)**[b]	σ^2 **(Å²)**[c]	**Δ*E*₀(eV)**	***R* factor**
Fe K-edge (S_0^2=0.777)						
Fe foil	Fe-Fe	8.0*	2.474±0.004	0.0051	7.4	0.0018
	Fe-Fe	6.0*	2.854±0.005	0.0066		
FeO	Fe-O	6.0±0.1	2.131±0.010	0.0121	4.8	0.0018
	Fe-Fe	12.0±0.3	3.049±0.004	0.0090	-2.5	
FeOOH	Fe-O	3.3±0.3	1.953±0.015	0.0025	-2.1	0.0105
	Fe-O	2.5±0.3	2.108±0.021			
	Fe-Fe	6.5±1.0	3.050±0.012	0.0092		
	Fe-Fe	6.5±1.5	3.293±0.024			
	Fe-Fe	6.3±1.8	3.449±0.024			
Fe_2O_3	Fe-O	3.8±0.2	1.940±0.011	0.0042	-2.6	0.0036
	Fe-O	2.4±0.3	2.108±0.018			
	Fe-Fe	4.8±0.2	2.957±0.007	0.0060	-1.2	
	Fe-Fe	3.7±0.3	3.386±0.009			
	Fe-Fe	4.9±0.3	3.691±0.007			
S-$LaNiFeCoCrMnO_3$	Fe-O	4.9±0.2	1.998±0.009	0.0103	-1.5	0.0179
	Fe-Fe	3.0±0.7	3.047±0.021	0.0178		
	Fe-Fe	3.1±1.0	3.428±0.028			
$LaNiFeCoCrMnO_3$	Fe-O	5.3±0.6	1.992±0.010	0.0131	-4.2	0.0033
	Fe-Fe	4.7±0.9	3.114±0.018	0.0195	4.7	
	Fe-Fe	3.6±0.4	3.429±0.018			
$LaNiFeCoCrO_3$	Fe-O	5.7±0.3	1.987±0.011	0.0111	-2.2	0.0131
	Fe-Fe	5.2±0.4	3.040±0.021	0.0211		
	Fe-Fe	4.9±0.8	3.477±0.027			

[a]*CN*, coordination number; [b]*R*, the distance between absorber and backscatter atoms; [c]σ^2, the Debye Waller factor value; [d]ΔE_0, inner potential correction to account for the difference in the inner potential between the sample and the reference compound; *R* factor indicates the goodness of the fit. $S0^2$ was fixed to 0.777, according to the experimental EXAFS fit of Fe foil by fixing *CN* as the known crystallographic value. * This value was fixed during EXAFS fitting, based on the known structure of Fe. Fitting conditions: *k* range : 2.0 - 10.0; *R* range: 1.0-4.0; fitting space: R space; *k*-weight = 3. A reasonable range of EXAFS fitting parameters: $0.800 < S_0^2 < 1.000$; $CN > 0$; $\sigma^2 > 0$ Å²; $|\Delta E_0| < 15$ eV; *R* factor < 0.02.

Table S4. EXAFS data fitting results of Fe K-edge for Fe foil and S-$LaNiFeCoCrMnO_3$ catalyst before stability test for the OER.

<table>
<tr><th>Samples</th><th>Path</th><th>CN^a</th><th>R(Å)^b</th><th>σ² (Å²)^c</th><th>ΔE₀(eV)</th><th>R factor</th></tr>
<tr><td colspan="7">Fe K-edge (S₀²=0.711)</td></tr>
<tr><td rowspan="2">Fe foil</td><td>Fe-Fe</td><td>8.0*</td><td>2.470</td><td>0.0046</td><td>6.9</td><td rowspan="2">0.0019</td></tr>
<tr><td>Fe-Fe</td><td>6.0*</td><td>2.846</td><td>0.0056</td><td>5.3</td></tr>
<tr><td rowspan="4">S-LaNiFeCoCrMnO₃</td><td>Fe-O</td><td>5.7</td><td>1.936</td><td>0.0094</td><td>-8.4</td><td rowspan="4">0.0179</td></tr>
<tr><td>Fe-Fe</td><td>1.8</td><td>2.728</td><td>0.0110</td><td>13.7</td></tr>
<tr><td>Fe-Ni</td><td>2.7</td><td>3.294</td><td rowspan="2">0.0053</td><td rowspan="2">2.4</td></tr>
<tr><td>Fe-Fe</td><td>2.2</td><td>4.085</td></tr>
</table>

Table S5. EXAFS data fitting results of Co K-edge for Co foil, CoO, and S-$LaNiFeCoCrMnO_3$ catalyst before stability test for the OER.

**Sample**	**Path**	***CN*[a]**	**R(Å)[b]**	**σ^2 ($Å^2$)[c]**	**ΔE_0(eV)**	**R factor**
Co K-edge (S_0^2=0.737)						
Co foil	Co-Co	12.0*	2.492±0.001	0.0061	7.5±0.2	0.0006
CoO	Co-O	5.8±0.4	2.100±0.006	0.0054	-4.0±0.7	0.0041
	Co-O-Co	12.3±0.3	3.010±0.005	0.0112		
S-LaNiFeCoCrMnO_3	Co-O	6.2±0.4	2.099±0.011	0.0205	6.1±0.7	0.0078
	Co-O-Co	4.9±0.5	3.254±0.009	0.0102		

[a]*CN*, coordination number; [b]*R*, the distance between absorber and backscatter atoms; [c]σ^2, the Debye Waller factor value; [d]ΔE_0, inner potential correction to account for the difference in the inner potential between the sample and the reference compound; *R* factor indicates the goodness of the fit. $S0^2$ was fixed to 0.737, according to the experimental EXAFS fit of Co foil by fixing *CN* as the known crystallographic value. * This value was fixed during EXAFS fitting, based on the known structure of Co. Fitting conditions: *k* range : 2.0 - 8.0; *R* range: 1.0-3.5; fitting space: R space; *k*-weight = 3. A reasonable range of EXAFS fitting parameters: $0.700 < S_0^2 < 1.000$; $CN > 0$; $\sigma^2 > 0$ $Å^2$; $|\Delta E_0| < 15$ eV; R factor < 0.02.

Table S6. EXAFS data fitting results of Ni K-edge for Ni foil and S-$LaNiFeCoCrMnO_3$ catalyst before stability test for the OER.

**Samples**	**Path**	CN^a	R(Å)^b	σ^2 (Å^2)^c	ΔE_0(eV)^d	**R factor**
Ni K-edge (S_0^2=0.806)						
Ni foil	Ni-Ni	12.0*	2.482	0.0061	7.6	0.0008
S-$LaNiFeCoCrMnO_3$	Ni-O	3.9	1.936	0.0055	-10.9	0.0157
	Ni-S	0.6	2.527	0.0088	0.4	
	Ni-Fe	2.3	3.353	0.0118	8.6	
	Ni-Ni	3.7	3.705			

Table S7. EXAFS data fitting results of Ni K-edge for Ni foil, NiOOH reference, NiO reference, $LaNiO_3$, $LaNiFeCoCrO_3$, $LaNiFeCoCrMnO_3$, and S-$LaNiFeCoCrMnO_3$ catalysts after stability test for the OER.

Samples	Path	CN^a	R(Å)^b	σ^2 (Å^2)^c	ΔE_0(eV)^d	R factor
Ni K-edge (S_0^2=0.807)						
Ni foil	Ni-Ni	12.0*	2.483±0.001	0.0061	7.7	0.0008
NiOOH	Ni-O	5.7±0.2	2.040±0.006	0.0090	-1.5	0.0083
	Ni-Ni	4.8±0.2	3.078±0.005	0.0078		
NiO	Ni-O	6.1±0.3	2.078±0.007	0.0051	-3.3	0.0055
	Ni-Ni	12.8±1.1	2.949±0.004	0.0059		
S-$LaNiFeCoCrMnO_3$	Ni-O	5.6±0.4	1.982±0.017	0.0165	-6.3	0.0140
	Ni-Ni	1.1±0.3	3.039±0.019	0.0155	10.3	
$LaNiFeCoCrMnO_3$	Ni-O	4.9±0.3	2.006±0.012	0.0113	-4.9	0.0149
	Ni-Ni	1.6±0.3	3.047±0.034	0.0192		
$LaNiFeCoCrO_3$	Ni-O	4.6±0.3	2.011±0.012	0.0120	-5.5	0.0189
	Ni-Ni	1.5±0.3	3.071±0.026	0.0162		
$LaNiO_3$	Ni-O	3.9±0.2	2.020±0.011	0.0089	-4.3	0.0129
	Ni-Ni	1.3±0.3	3.076±0.019	0.0110		

^{a}*CN*, coordination number; ^{b}*R*, the distance between absorber and backscatter atoms; $^c\sigma^2$, the Debye Waller factor value; $^d\Delta E_0$, inner potential correction to account for the difference in the inner potential between the sample and the reference compound; *R* factor indicates the goodness of the fit. $S0^2$ was fixed to 0.807, according to the experimental EXAFS fit of Ni foil by fixing *CN* as the known crystallographic value. * This value was fixed during EXAFS fitting, based on the known structure of Ni. Fitting conditions: *k* range : 3.0 - 11.0; *R* range: 1.0-3.0; fitting space: R space; *k*-weight = 3. A reasonable range of EXAFS fitting parameters: $0.800 < S_0^2 < 1.000$; $CN > 0$; $\sigma^2 > 0$ Å^2; $|\Delta E_0| < 15$ eV; *R* factor < 0.02.

References

[1] G.Kresse, J. Hafner, *Phys. Rev. B* 1994, **47**, 14251–1426

[2] E. P. E. Blöchl, Phys. Rev. B 1994, **50**, 17953.

[3] J. P. Perdew, K. Burke, M. Ernzerhof, *Phys. Rev. Lett.* 1996, **77**, 3865–3868.

[4] S. Grimme, J. Antony, S. Ehrlich, H. Krieg, *J. Chem. Phys.* 2010, **132**, 154104.

[5] V. I. Anisimov, F. Aryasetiawan, A. J. Lichtenstein, *J. Phys. Condens. Matter.* 1997, **9**, 767.

[6] D. Friebel, M. W. Louie, M. Bajdich, K. E. Sanwald, Y. Cai, A. M. Wise, M.-J. Cheng, D. Sokaras, T.-C. Weng, R. Alonso-Mori, R. C. Davis, J.R. Bargar, J. K. Nørskov, A. Nilsson, A. T. Bell, *J. Am. Chem. Soc.* 2015, **137**, 1305.

[7] H. J. Monkhorst, J. D. Pack, Special points for Brillouin-zone integrations. *Phys. Rev. B*, 1976, **13**, 5188−5192.

[8] V. L. Deringer, A. L. Tchougréeff and R. Dronskowski, Crystal Orbital Hamilton Population (COHP) Analysis as Projected from Plane-Wave Basis Sets, *J. Phys. Chem. A*, 2011, **115**, 5461–5466.

[9] T. X. Nguyen, Y. H. Su, C. C. Lin, J. M. Ting, *Adv. Funct. Mater.*, 2021, **31**, 2106229.

[10] X. Zhang, Y. Yang, Y. Liu, Z. Jia, Q. Wang, L. Sun, L. C. Zhang, J. J. Kruzic, J. Lu, B. Shen, *Adv. Mater.*, 2023, **35**, 2303439.

[11] T. Wang, J. Fan, C. L. Do-Thanh, X. Suo, Z. Yang, H. Chen, Y. Yuan, H. Lyu, S. Yang, S. Dai, *Angew. Chem., Int. Ed.,* 2021, **60**, 9953-9958.

[12] H. Shi, X. Y. Sun, Y. Liu, S. P. Zeng, Q. H. Zhang, L. Gu, T. H. Wang, G. F. Han, Z. Wen, Q. Fang, X. Y. Lang, Q. Jiang, *Adv. Funct. Mater.* 2023, **33**, 2214412.

[13] Y. Wang, X. Ge, Q. Lu, W. Bai, C. Ye, Z. Shao, Y. Bu, *Nat. Commun.,* 2023, **14**, 6968.

[14] Y. Hu, Y. Zheng, J. Jin, Y. Wang, Y. Peng, J. Yin, W. Shen, Y. Hou, L. Zhu, L. An, M. Lu, P. Xi, C. H. Yan, *Nat. Commun.*, 2023, **14**, 1949.

[15] E. Fabbri, M. Nachtegaal, T. Binninger, X. Cheng, B. J. Kim, J. Durst, F. Bozza, T. Graule, R. Schaublin, L. Wiles, M. Pertoso, N. Danilovic, K. E. Ayers, T. J. Schmidt, *Nat. Mater.*, 2017, **16**, 925-931.

[16] B. Q. Li, Z. J. Xia, B. Zhang, C. Tang, H. F. Wang, Q. Zhang, *Nat. Commun.,* 2017, **8**, 934.

[17] Y. Tang, C. Wu, Q. Zhang, H. Zhong, A. Zou, J. Li, Y. Ma, H. An, Z. Yu, S. Xi, J. Xue, X. Wang, J. Wu, *Angew. Chem., Int. Ed.* 2023, **62**, e202309107.

[18] N. Zhang, Y. Hu, L. An, Q. Li, J. Yin, J. Li, R. Yang, M. Lu, S. Zhang, P. Xi, C. H. Yan, *Angew. Chem., Int. Ed.*, 2022, **61**, e202207217.

[19] H. Wang, J. Wang, Y. Pi, Q. Shao, Y. Tan, X. Huang, *Angew. Chem., Int. Ed.*, 2019, **58**, 2316-2320.

[20] Y. Yang, H. Yao, Z. Yu, S. M. Islam, H. He, M. Yuan, Y. Yue, K. Xu, W. Hao, G. Sun, H. Li, S. Ma, P. Zapol, M. G. Kanatzidis, *J. Am. Chem. Soc.*, 2019, **141**, 10417-10430.

[21] T. Wang, H. Chen, Z. Yang, J. Liang, S. Dai, *J. Am. Chem. Soc.*, 2020, **142**, 4550-4554.

[22] X. Ren, Y. Zhai, P. Wang, Z. Xu, S. Gao, X. Chen, Q. Gu, B. Wang, J. Li, S. F. Liu, *Adv. Mater.*, 2023, **35**, 2301166.

[23] A. Wang, X. Zhang, S. Gao, C. Zhao, S. Kuang, S. Lu, J. Niu, G. Wang, W. Li, D. Chen, H. Zhang, X. Zhou, S. Zhang, B. Zhang, W. Wang, *Adv. Mater.*, 2022, **34**, 2204247.

[24] T. Dai, X. Zhang, M. Sun, B. Huang, N. Zhang, P. Da, R. Yang, Z. He, W. Wang, P. Xi, C. H. Yan, *Adv. Mater.*, 2021, **33**, 2102593.

[25] P. Ye, K. Fang, H. Wang, Y. Wang, H. Huang, C. Mo, J. Ning, Y. Hu, *Nat. Commun.*, 2024, **15**, 1012.

[26] B. Zhang, X. Zheng, O. Voznyy, R. Comin, M. Bajdich, M. García-Melchor, L. Han, J. Xu, M. Liu, L. Zheng, F. P. García de Arquer, C. T. Dinh, F. Fan, M. Yuan, E. Yassitepe, N. Chen, T. Regier, P. Liu, Y. Li, P. De Luna, A. Janmohamed H. L. Xin, H. Yang, A. Vojvodic, E. H. Sargent, *Science* 2016, **352**, 333–337.

[27] J. Cai, H. Zhang, L. Zhang, Y. Xiong, T. Ouyang, Z. Q. Liu, *Adv. Mater.*, **2023**, *35*, e2303488.

[28] W. Hooch Antink, S. Lee, H. S. Lee, H. Shin, T. Y. Yoo, W. Ko, J. Shim, G. Na, Y. E. Sung, T. Hyeon, *Adv. Funct. Mater.*, 2024, **34**, 2309438.

[29] Z. Y. Yu, Y. Duan, Y. Kong, X. L. Zhang, X. Y. Feng, Y. Chen, H. Wang, X. Yu, T. Ma, X. Zheng, J. Zhu, M. R. Gao, S. H. Yu, *J. Am. Chem. Soc.*, 2022, **144**, 13163-13173.

[30] X. Li, H. Liu, Z. Chen, Q. Wu, Z. Yu, M. Yang, X. Wang, Z. Cheng, Z. Fu, Y. Lu, *Nat. Commun.*, 2019, **10**, 1409.

[31] T. Wu, Y. Sun, X. Ren, J. Wang, J. Song, Y. Pan, Y. Mu, J. Zhang, Q. Cheng, G. Xian, S. Xi, C. Shen, H. J. Gao, A. C. Fisher, M. P. Sherburne, Y. Du, J. W. Ager, J. Gracia, H. Yang, L. Zeng, Z. J. Xu, *Adv. Mater.*, 2023, **35**, 2207041.

[32] S. Luo, C. Dai, Y. Ye, Q. Wu, J. Wang, X. Li, S. Xi, Z. J. Xu, *Angew. Chem., Int. Ed.*, 2024, **63**, e202402184.

[33] S. Li, T. Liu, W. Zhang, M. Wang, H. Zhang, C. Qin, L. Zhang, Y. Chen, S. Jiang, D. Liu, X. Liu, H. Wang, Q. Luo, T. Ding, T. Yao, *Nat. Commun.*, 2024, **15**, 3416.
[34] X. Bai, M. Zhang, Y. Shen, X. Liang, W. Jiao, R. He, Y. Zou, H. Chen, X. Zou, *Adv. Funct. Mater.*, **2024**, 34, 2400979.
[35] L.-W. Shen, Y. Wang, L. Shen, J.-B. Chen, Y. Liu, M.-X. Hu, W.-Y. Zhao, K.-Y. Xiong, S.-M. Wu, Y. Lu, J. Ying, M. M. Titirici, C. Janiak, G. Tian, X.-Y. Yang, *Energy Environ. Sci.*, 2024, **17**, 3888-3897.
[36] L. Wan, J. Liu, D. Lin, Z. Xu, Y. Zhen, M. Pang, Q. Xu, B. Wang, *Energy Environ. Sci.*, 2024, **17**, 3396-3408.
[37] L. Wan, Z. Xu, Q. Xu, P. Wang, B. Wang, *Energy Environ. Sci.*, 2022, **15**, 1882-1892.
[38] P. Thangavel, M. Ha, S. Kumaraguru, A. Meena, A. N. Singh, A. M. Harzandi, K. S. Kim, *Energy Environ. Sci.*, 2020, **13**, 3447-3458.
[39] A. Nairan, Z. Feng, R. Zheng, U. Khan, J. Gao, *Adv. Mater.*, **2024**, 36, 2401448.
[40] A. Nairan, Z. Feng, R. M. Zheng, U. Khan, J. K. Gao, *Adv. Mater.* **2024**, *36*, 2401448.
[41] G. Zhang, J. Pei, Y. Wang, G. Wang, Y. Wang, W. Liu, J. Xu, P. An, H. Huang, L. Zheng, J. Dong, J. Zhang, *Angew. Chem., Int. Ed.*, 2024, **63**, e202407509.
[42] L. Zong, F. Lu, P. Li, K. Fan, T. Zhan, P. Liu, L. Jiang, D. Chen, R. Zhang, L. Wang, *Adv. Mater.*, 2024, **36**, 2403525.
[43] J. Wang, C. Liang, X. Ma, P. Liu, W. Pan, H. Zhu, Z. Guo, Y. Sui, H. Liu, L. Liu, C. Yang, *Adv. Mater.*, **2**024, **36**, 2307925.
[44] J. Abed, S. Ahmadi, L. Laverdure, A. Abdellah, C. P. O'Brien, K. Cole, P. Sobrinho, D. Sinton, D. Higgins, N. J. Mosey, S. J. Thorpe, E. H. Sargent, *Adv. Mater.*, 2021, **33**, 2103812.
[45] Z. Li, G. Lin, L. Wang, H. Lee, J. Du, T. Tang, G. Ding, R. Ren, W. Li,X. Cao, S. Ding, W. Ye, W. Yang, L. Sun, *Nat. Catal.* 2024, **7**, 944
[46] A. C. Garcia, T. Touzalin, C. Nieuwland, N. Perini, M. T.Koper, *Angew. Chem. Int. Ed.* 2019, **58**, 12999–13003
[47] G. Deng, Y. Liao, Y. Lin, L. Ding, H. Wang, *Angew. Chem. Int.Ed.* 2024, **63**, e202412632.
[48] L. Yin, R. Ren, L. He, W. Zheng, Y. Guo, L. Wang, H. Lee, J. Du, Z. Li, T. Tang, G. Ding, L. Sun, *Angew. Chem. Int. Ed.* 2024, **63**, e202400764.
[49] M. Klingenhof, H. Trzesniowski, S. Koch, J. Zhu, Z. Zeng, L. Metzler, A. Klinger, M. Elshamy, F. Lehmann, P. W. Buchheister, A. Weisser, G. Schmid, S. Vierrath, F. Dionigi, P. Strasser, High-performance anion-exchange membrane water electrolysers using NiX (X = Fe,Co,Mn) catalyst-coated membranes with redox-active Ni-O ligands. *Nat. Catal.* 2024, **7**, 1213.
[50] X. Cui, T. Tang, F. Zhang, L. Sun, B. Zhang, New benchmark for pure nickel-based oxygen-evolution electrocatalyst: Tailored large $NiMoO_4 \cdot xH_2O$ monocrystals for complete reconstruction. *Appl. Catal. B: Environ.* 2025, **366**, 125024.
